# Visual sequence encoding is modulated by music schematic structure and familiarity

**DOI:** 10.1371/journal.pone.0306271

**Published:** 2024-08-07

**Authors:** Yiren Ren, Grace Leslie, Thackery Brown

**Affiliations:** 1 School of Psychology, Georgia Institute of Technology, Atlanta, GA, United States of America; 2 ATLAS Institute, University of Colorado Boulder, Boulder, CO, United States of America; Federal University of Paraiba, BRAZIL

## Abstract

Music is omnipresent in daily life and may interact with critical cognitive processes including memory. Despite music’s presence during diverse daily activities including studying, commuting, or working, existing literature has yielded mixed results as to whether music improves or impairs memory for information experienced in parallel. To elucidate how music memory and its predictive structure modulate the encoding of novel information, we developed a cross-modal sequence learning task during which participants acquired sequences of abstract shapes accompanied with paired music. Our goal was to investigate whether familiar and structurally regular music could provide a “temporal schema” (rooted in the organized and hierarchical structure of music) to enhance the acquisition of parallel temporally-ordered visual information. Results revealed a complex interplay between music familiarity and music structural regularity in learning paired visual sequences. Notably, compared to a control condition, listening to well-learned, regularly-structured music (music with high predictability) significantly facilitated visual sequence encoding, yielding quicker learning and retrieval speed. Conversely, learned but irregular music (where music memory violated musical syntax) significantly impaired sequence encoding. While those findings supported our mechanistic framework, intriguingly, unlearned irregular music–characterized by the lowest predictability–also demonstrated memory enhancement. In conclusion, this study demonstrates that concurrent music can modulate visual sequence learning, and the effect varies depending on the interaction between both music familiarity and regularity, offering insights into potential applications for enhancing human memory.

## Introduction

Music has been deeply ingrained in daily life from the early stages of human history [1, 2]. It is universally enjoyed, and humans demonstrate remarkable, perhaps innate abilities in music acquisition and perception [3–5]. Because of this, with the easy access to music in modern society (e.g., via Bluetooth), it is frequent to see people listening to music while doing other things, including important tasks like studying, driving, or working. This phenomenon has led researchers to question whether music could be beneficial for memory function. Prior studies have provided evidence that music, especially when familiar, can enhance memory recall and language-related learning [6–10]. For example, data have shown that listening to familiar music can evoke past memories, and this mechanism has been used to help cue more vivid autobiographical memory recall in Alzheimer’s patients [7, 11]. Others have found music pairing improved verbal memory and language learning, potentially due to overlap between music processing mechanisms and our language system [8, 9]. However, studies about whether music improves or detracts from memory *encoding* for non-musical, non-linguistic information have shown mixed results–e.g., one study showed that pop music played during learning images hindered immediate recall while another found no influence of novel music on verbal learning [12, 13]. One plausible explanation is rooted in the fact that music is itself a complex multidimensional event (taxing spatiotemporal memory, to the extent tones rise and fall in complex and often layered patterns over time). Instead of expecting music to have a consistent single effect on learning non-music information, here we test the hypothesis that the structure of memory-based music characteristics can induce varying effects on the encoding processes of non-musical, non-linguistic memory. Specifically, we constrained this broad topic to target whether different characteristics of music that are dependent on memory (familiarity with a specific composition, and how well the structural composition follows schematic memory–the “language” of music), modulate encoding of parallel sequences of visual items.

The ability to predict upcoming stimuli plays an essential role in human decision-making and in rendering memory more efficient. For example, in the psychology of memory, “schema theory” holds that for associative memories, new learning that is congruent with the structure of existing schemas is encoded and consolidated easier and faster (putatively because existing associations help bridge the new associations to be formed) [14–16]. Similarly, humans show fast music learning skills due to a powerful interconnected music schema system–that is, the memories of different music share basic patterns that allow for expectations of future tones during novel music listening [17]. Such music expectations are based on both listeners’ veridical experiences (knowing the specific music composition) and schematic music structural knowledge (musical syntax) [18, 19]. Moreover, these two sources of expectation might work independently and sometimes even compete with each other, supported by both behavioral and neuroscience data [18, 19–21].

Notably, prior literature has tested the use of musical expectancy to guide attention during cognitive tasks of other domains, such as visual encoding (e.g. faces) or time/duration judgments [22, 23]. and has suggested sound and music could provide an “auditory scaffolding” for new sequence learning [24]. This benefit is putatively by providing a more regular and clear temporal context cues for the associated memory (e.g., when an given note transition occurs, one can better remember the relative ordinal position in the sequence, how much time has elapsed since the last stimulus in a sequence one is learning, and/or when a next associated stimulus may be expected). One relevant example is the “alphabet song” that helps babies to learn language during early stage. This theoretical background prompted us to question whether music, with its sequences of tones and rhythms governed by language-like structural rules, could provide a sequential context that can modulate how new information, encoded in parallel, becomes itself associated in sequence. If so, considering that prior studies suggest schema effects on new learning can be multi-dimensional (e.g., prior knowledge of specific pairing between visual information about face and medical diagnosis can improve acquisition and generalization of more abstract medical knowledge) [15, 25], this benefit from music should extend beyond sequencing new auditory stimuli to also apply to sequencing non-auditory stimuli. In this way, music might provide a template to help human encode series of events more generally. One similar scenario is using music to support encoding a series of words (as some advertisements do). In this study, we aimed to investigate the effect of music on the temporal learning of non-textual information ‐ namely abstract shapes–and to uncover the importance of two dimensions of music predictability for these effects.

To investigate this question, we devised a novel cross-modal sequential memory task, bridging visual sequence memory and music listening. A recent study tested cross-modal sequence learning by a utilizing motor sequence to aid human’s visual item series encoding [26]. Our study tested a similar idea, but using music (auditory sequence). In this task, we paired unfamiliar abstract visual stimuli with music of varying degrees of predictability during the visual sequence memory encoding phase. Inspired by music literatures suggesting two separate mechanisms for music expectancy [18, 27], in this study, we separately defined and manipulated these two types of predictability as *music familiarity* (knowing the music veridically) and *music regularity* (syntactical correctness according to the schemas listeners have for how music is temporally and tonally structured and unfolds over time), and we tested how they together affected parallel visual sequential learning.

**Fig 1:**
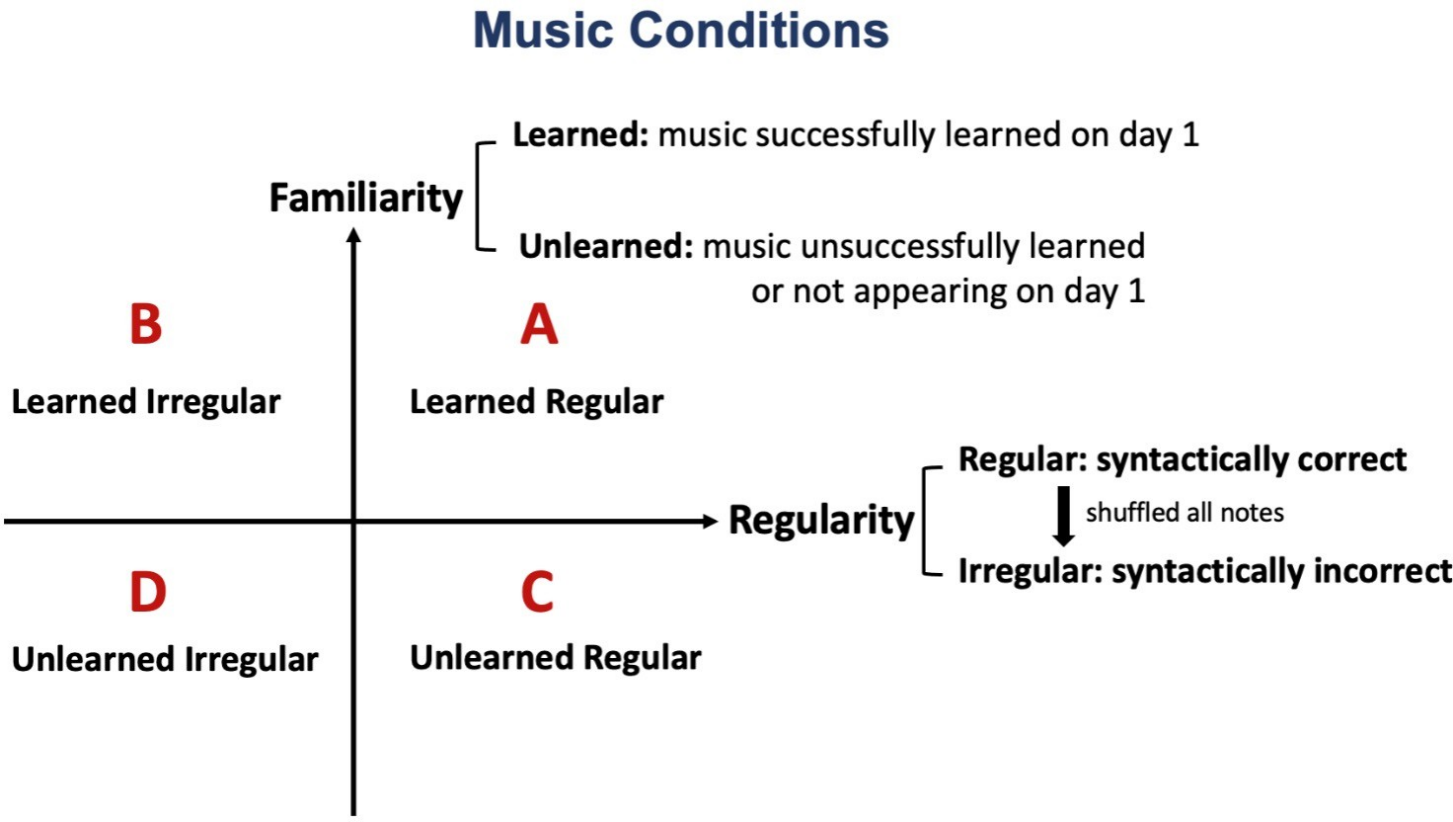
Music stimulus conditions. To test how music expectancy modulates parallel visual learning, we implemented a factorial design across two experimental variables–music familiarity and music regularity. Each variable had two conditions, making four music conditions in total. A: learned regular music; B: learned irregular music; C: unlearned regular music, D: unlearned irregular music.

Shown in Fig 1, to achieve this, we had two conditions of music **familiarity**–*learned* and *unlearned* music, implemented by a music training task a day prior visual sequences learning (see Method and Results for details). We also manipulated music in the level of its structural and syntactical correctness and referred to it as music **regularity**. Past studies showed that music with a regular pattern, congruent with the Western Classical syntax of the stimuli we used, is predictable even for the first time listening [19, 28]. Prior work has shown that compared to syntactically regular music, irregular or unstructured musical sequences elicit differential neural response in multiple brain regions, associating with processing prediction errors, sequence regularity processing and emotion [29–32]. Some studies have shown that people have better working memory and long-term memory for music that follows syntax (tonal music) [33, 34]. Such data, together with the schema theory framework, emphasize that humans are equipped with predictive coding for syntax-correct music based on prior knowledge of other compositions and music’s “language” alone.

Reflecting on the variability in prior literature on how music affects different types of memory [35, 36], we hypothesized that varied results might have been influenced by many studies using music with freely varying parameters across dimensions such as regularity, familiarity, as well as timbre and music style. The current literature has used highly varied methodologies and memory measures, employing both single-day [e.g. 37] and multi-day designs [e.g. 9] to test how background music influences learning and retrieval. One important design difference from the current study is that commonly, studies with background music have presented it unsynchronized with the primary information to be learned. Importantly, this is a common naturalistic scenario. But, it might encourage participants to perceive it a backdrop distractor, could explain the mixed reports on its effects on memory [38, 39]. Designs that have more reliably shown benefits on memory often involve synchronized music. This is typically done with text-based or verbal memory tasks [40, 41], potentially integrating the text as a part of the music. However, evidence for how music influences visual memory is surprisingly limited. Our study aimed to fill this gap by synchronizing the onset of music with the presentation of visual stimuli, which were abstract to avoid existing semantic associations conflicting with influences of the music properties we manipulated. This method encouraged active engagement with the music when learning rather than treating it as background noise, addressing a potential applied angle for this research to visual learning problems. Additionally, most prior studies used pre-existing music (e.g., popular music or classic music) which varies in complexity and familiarity [38, 42]. We were inspired by one study that looked at music’s effect on verbal memory composed their own music to ensure the stimuli were unknown and had varied tempo and consonance [13]. To explore how predictable features of music structure affect memory and whether visual sequential associations are influenced similarly to text-based memory, we needed to develop a new design. Here, we manipulated regularity and familiarity in truly novel experimentally-generated music, which allowed us to understand music expectancy’s effect on cross-modal memory encoding without lifetime familiarity and autobiographical associations with the music confounding the results. We believe this approach offers insights which can bridge the existing gap between literature on music cognition and other memory research. These insights are particularly relevant for endeavors aimed at enhancing memory efficiency in an applied setting.

Motivated by the literature reviewed above on schemas in both music and non-music settings, we hypothesized that listening to a piece of highly predictable music (Condition **A–***learned regular*, Fig 1), which is both familiar and regular to the listeners, could benefit learning of new arbitrary sequences of information that are paired with such music by providing more informative and robust representation of auditory sequences as a “temporal context” backdrop to the new learning (i.e., aid associations between myriad features of our lives from a variety of modalities; here we used novel abstract shape sequences as a proxy to test this idea because they are of a different modality). Conversely, we hypothesized that music that was less syntactically regular and less familiar (Condition **D–***unlearned irregular*, Fig 1) would not help or could even interfere with parallel visual sequential learning. One straightforward mechanistic explanation for such a detrimental effect could lie in evidence suggesting compared to regular music, syntactically incorrect music leads to prediction errors and generate unpleasantness, potentially occupying cognitive load and leading to more interferences with concurrent cognitive task [43, 44]. One interesting result would be the interaction between music familiarity and regularity. Past literature suggests a competing relationship between veridical and schematic expectancy in music [18]. As a result, we expected reduced improvement or even disruptive effects when the music is familiar but irregular, or vice versa (Condition **B** or **C** on Fig 1).

## Materials and methods

### Participants

Fifty-one participants were recruited from the Georgia Institute of Technology’s volunteer pool. One participant withdrew during the task, and two participants were excluded from the analysis due to a lack of responses during the task, leaving 48 participants aged 18–24 years (25 females, 23 males). Participants were pre-screened via self-report questionnaire to exclude individuals with the following conditions: hearing problems, abnormal uncorrected vision, basic music recognition problems such as amusia or music agnosia, learning disability, attention disability, or history of other neurological or psychiatric disorders. All experiments were carried out in accordance with the guidelines and regulations provided by the Institutional Review Board of Georgia Institute of Technology. All experimental protocols were approved by the Institutional Review Board of Georgia Institute of Technology. All participants gave the written informed consent before participation. Our recruitment started on March 4^th^, 2020 and ended on November 12^th^, 2020.

All participants finished a survey regarding their music background history. Twelve of the participants had no prior music training experiences. We asked which musical instrument(s) they had learned for at least one year, and how long they studied and practiced each instrument. The average cumulative years of musical instrument training for participants with prior music training was 11.59 years (min = 2, max = 23, s.d. = 6.15, median = 8).

### Experimental materials

#### Music stimuli

Data and study materials are available online: https://osf.io/frxdz/. All music stimuli were composed manually in Logic Pro X (https://www.apple.com/logic-pro/) based on the following principles: To test the effect on visual sequence memory of music with different levels of regularity and syntactic structure, we created three auditory conditions (12 audio clips in each condition).

The reference (control) condition was made up of "compositions" where each piece of music/audio (12 in total) was an isochronous tone sequence–a single note (chosen from notes C_3_ to C_6_) was played eight times at a steady interval, once per second using a single instrument (piano, clarinet or flute), making each clip eight seconds. We chose a monotonic sequence as an active control because it ensured that results from our two experimental conditions could not be attributed to the simple presence of sound during visual encoding. Technically, the control condition falls along a music continuum with our other conditions, being predictable given its simple temporal structure, but lacks the musical dynamics and hierarchical tonal-rhythmic features of true music.

By contrast, in the experimental conditions, the music was an 8-second mono-layer melody, each played by a single instrument (piano, clarinet or flute), with a tempo of 60 beats per minute. In this way, our experimental conditions resemble the context where a piece of music might be played in an advertisement paired with a slogan–brief but musical and memorable. We opted to use monotonic streams as control, instead of no music, in our design for the compatibility of this design with our planned follow-up MRI study (in preparation at this time of writing), where it was important for several reasons that all conditions be matched in having auditory input.

1. In the *Regular* condition (12 in total), the music compositions had an organized and predictable tonal and temporal structure within a specific key. The pitches of each regular music melody implied a chord progression following Western Classical Music syntax (we readily acknowledge the eurocentricity of this operationalization and do not imply a judgment of Western Classical vs. non-Western Classical music).
2. The *Irregular* condition (12 in total) was made of regular pieces of music composed on the same principles as above, which were then transformed by pseudo-randomizing the sequence of the notes and the temporal intervals between notes that occur. This changed the temporal occurrence of each note and thus–in contrast to the regular condition–the melody would not imply a chord progression and the temporal interval between notes did not follow the typical "power of two" divisions standard in Western Classical Music. However, the keys belonging to one piece of irregular condition music were still in octave relationship and thus were not dissonant. Because of this, the irregular music was not auditory "noise"–it did sound broadly like a music composition and each composition was still distinct. But, critically for our study, it no longer retained the musical syntax: a regular temporal structure and the predictability characteristic of the regular/tonal condition. See Fig 3C for example notes for regular versus irregular music stimuli.

#### Music stimulus validation

**Fig 2:**
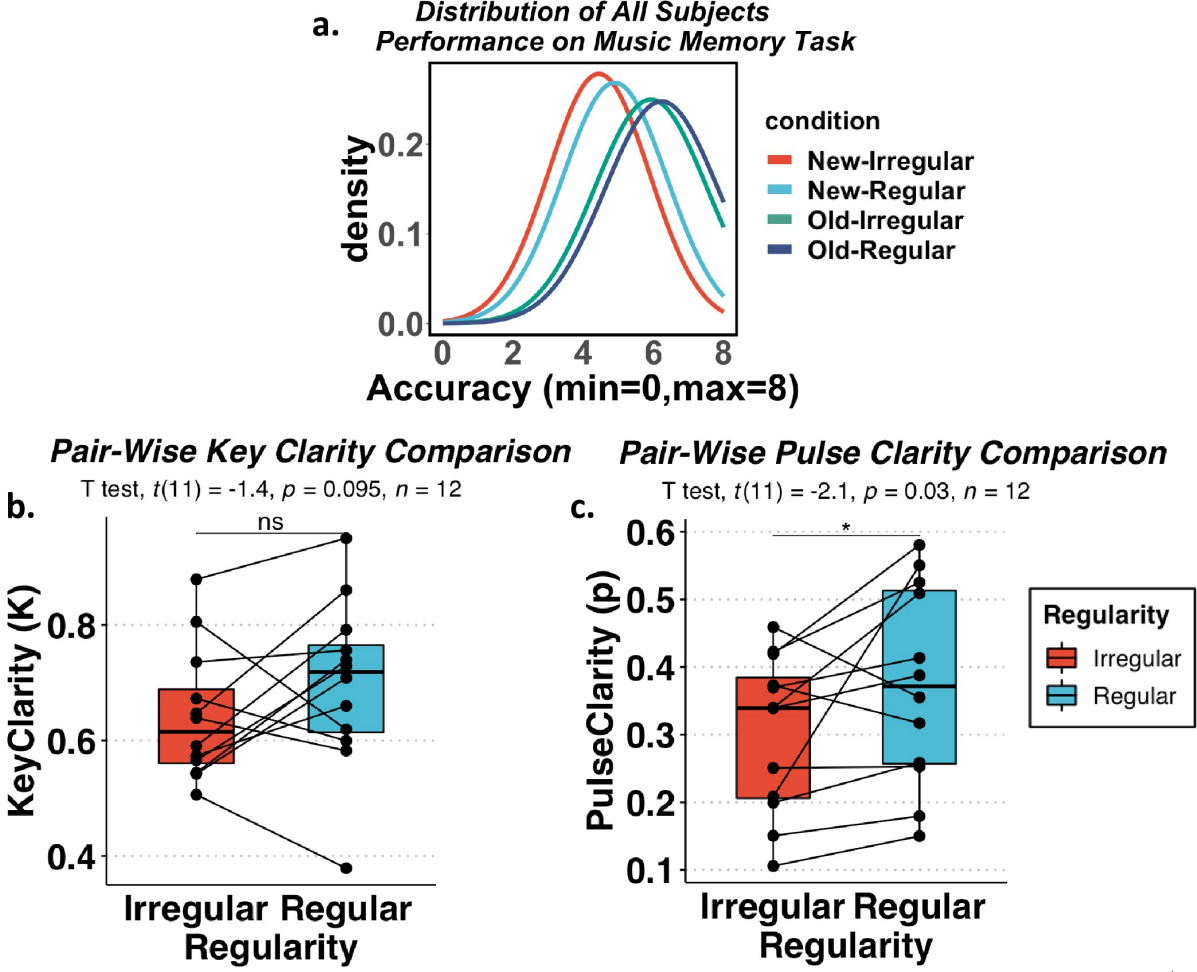
Music learning and music condition validation. **(A)** Final music memory retrieval performance (36 music stimuli in total): The task returned a retrieval score from 0–8. The plot shows the accuracy distribution for each music condition (excluding the control condition, which cannot have errors/be tested). (**B) (C)** key clarity and pulse clarity were measured using MIRtoolbox from MATLAB for both *regular* and *irregular* stimuli. Pair-wise comparisons (t-test) were conducted to compare *regular* and *irregular* music in these two features. Each pair of *regular* and *irregular* music is connected using lines. T-tests showed significantly higher pulse clarity in *regular* music but only a trend difference in clarity.

Our music stimuli were composed manually by a Western Classical music-trained musician. Because the music compositions were not generated synthetically (e.g., by a music-producing algorithm), it was important to verify whether the syntactical and hierarchical structure did differ between *regular* and *irregular* conditions and ask what features of them did we change when we scrambled the notes in *regular* condition. Thus we computed the statistical properties of the music and validated the intended structural differences of the *regular* and *irregular* stimuli used in this study using quantitative means provided by the MIRtoolbox (Music Information Retrieval) from MATLAB [45]. Past music studies have tested this toolbox in its ability to distinguish and analyze instrumental *regular* and *irregular* music, as defined in the present study, with these studies highlighting the *key clarity* and *pulse clarity* functions (quantifying the clarity and distinctness of the main key and pulse pattern of the music) as critical properties for music regularity [46, 47]. We thus used MIRtoolbox and computed the key clarity and pulse clarity of all music (excluding control) and ran pair-wise comparisons (since *irregular* music was generated from a regular music counterpart.). In Fig 2B and 2C, we demonstrated that the pulse clarity in our *regular* music stimuli was significantly greater than in our *irregular* music stimuli (t_11_ = -2.1, p = .03) on average across pairs. There was no significant difference between the key clarity of *regular* and *irregular* music (t_11_ = -1.4, p = .095), which might due to 1) small sample size and 2) the fact that both music compositions shared the same notes, and even in the *irregular* music notes were correlated with each other across time and belonged to the same key. As a result, the main difference between our *regular* and *irregular* stimuli lies in the regularity of the underlying beat or rhythmic pulse of the music. In our stimuli, the temporal intervals between tones in irregular music became less stable and less predictable. For example, when a birthday song is shuffled the way we did to create the stimuli (here is a link to an irregular birthday song: https://osf.io/nm9sg), the timing of the notes became less regular than before. As an analog, in human language, the words of an "irregular" sentence would be put in an order that did not follow a syntax predicted by the listener and thus would not make sense–each word would be clear and comprehensible, but a meaningful structural relationship between them was lacking for higher-level linguistic meaning.

#### Visual stimuli

**Fig 3:**
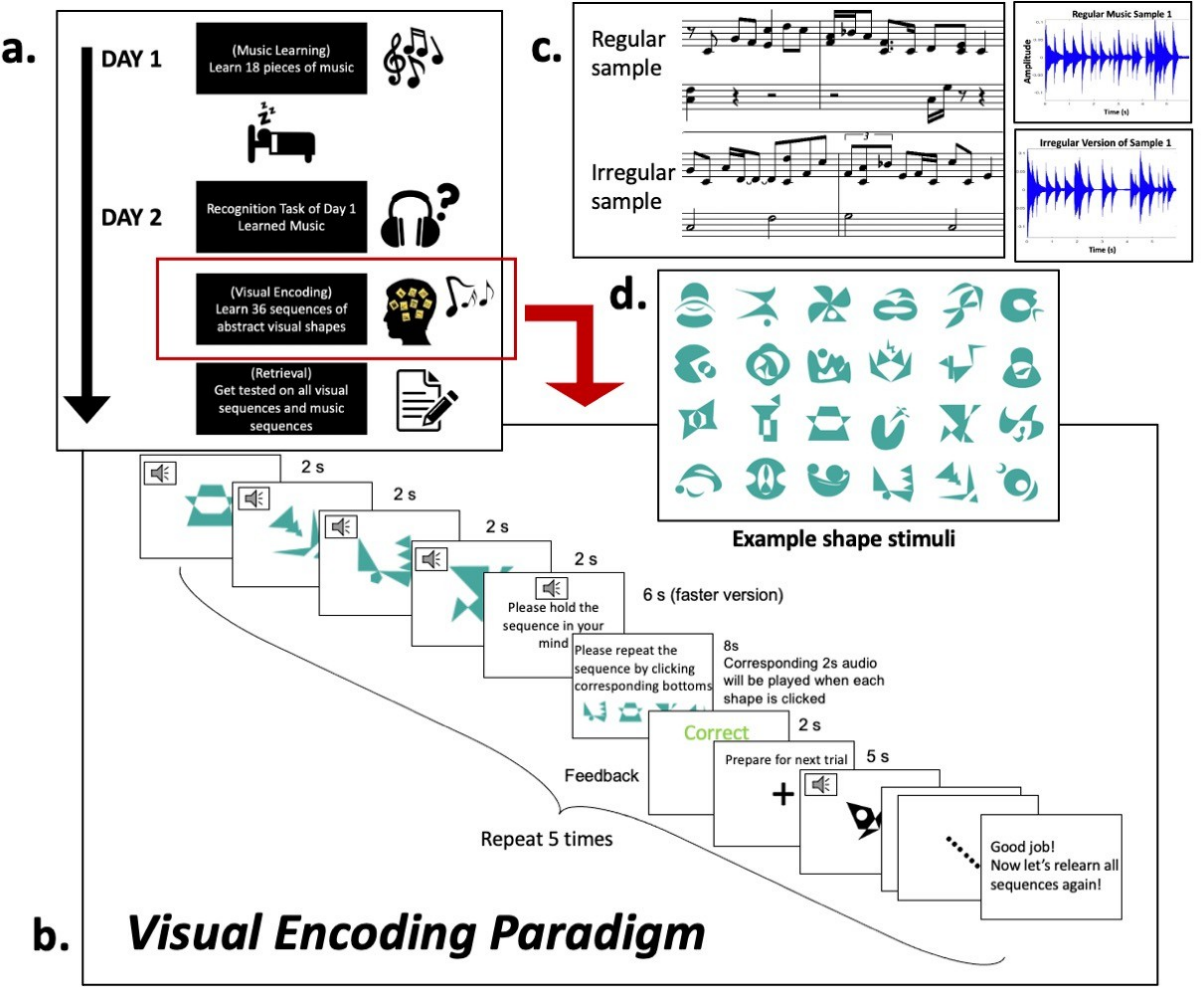
Experiment paradigm. **(A)** Two-day task procedure **(B)** Visual encoding paradigm used on Day 2: this is the main task phase during which participants repeatedly learned visual sequences paired with music. **(C)** notes and waveforms for example *regular* music stimuli and the *irregular* version of it. **(D)** example visual shape stimuli.

The *visual stimuli*: The critical test of our hypotheses came through differences in how people learned visual sequences paired with the music stimuli described above. To minimize semantic associations modulating the visual sequence learning as an uncontrolled factor, we generated arbitrary visual sequences from novel abstract shapes manually made using GNU Image Manipulation Program (https://www.gimp.org/). Each abstract and irregular shape was made up of either simple lines or curves. These shapes were not overly-simple (compared to simple shapes such as triangle or square) because we wanted people to be able to recognize and differentiate them (since there were 36 sequences of 4 shapes– 144 different shapes overall (example shapes in Fig 3D)) but do so without any systematic prior semantic knowledge of their identity (i.e., task novel stimuli, like the music stimuli were).

### Experiment procedures

We used a two-day experiment containing three parts: music learning, visual encoding, and retrieval. All tasks were delivered using Psychtoolbox3 (www.psychotoolbox.org). In brief, (shown in Fig 3A) the participants learned half of the music stimuli during the *music learning* phase on Day 1. On the second day, during the *visual encoding* phase, they learned novel abstract shape sequences paired with *learned* and *unlearned* music. In the end, during the *retrieval* phase, a test was given on the memory of the visual sequences without any auditory cues.

#### Music learning phase (Day 1)

The main goal for this phase was to implement our manipulation of music familiarity. This stage was accomplished the day before visual stimulus sequence learning so that the participants had a sleep cycle to help consolidate the memory of music. Participants were asked to memorize 18 novel music stimuli explained above (with six randomly sampled from the 12 regular compositions, six randomly sampled from the 12 irregular compositions, and six randomly from the 12 control sequences). Motivated by behavioral piloting, the music learning task contained two parts to achieve good learning in a broad range of participants–in the first part, the participants listened passively to all 18 music sequences in loops until they felt they had memorized each music composition. Once they finished passively learning all the music, they then proceeded to a music retrieval task to solidify participants’ knowledge of the composition through retrieval and practice.

In this retrieval task (see S1 Fig), the compositions were divided into six parts for each trial. The participant would first listen to the first 2 second of a piece of previously encountered audio, followed by five consecutive auditory recognition choice questions for the remaining parts of the composition. For each of the five rest parts of the audio after the initial music memory cue, the correct music fragment was presented along with a luring choice for the participant to listen to. The participant needed to re-compose the music by choosing the correct piece for each part and filling in all five blanks/parts for that composition. This test effectively asked the participant to practice/play the music using provided chunks of the musical pieces (accommodation for the fact that we could not ask them to reproduce the music with, for example, a digital keyboard, particularly in cases where the participant would not have prior musical instrument training).

For participants to continue learning from this phase, when the participant made any mistakes on the retrieval/practice task, the participant would hear the correct music again in a loop until they pressed a key indicating that they knew the piece. After all 18 compositions had been tested in one run, the participant was tested again on the next run, specifically on the compositions they had made mistakes in the prior run. Across runs, once the participant correctly reproduced a specific music composition on a retrieval task repetition with no mistakes, that music sequence would be marked as “successfully learned” and would not be tested again in subsequent runs. Notably, for the monotonic control condition, this re-composition task is trivial because the participants simply needed to select the same tone of the first 2 second. As a result, all monotonic clips were technically ‘learned’ on Day 1 and were categorized this way solely to create a one-to-one comparison with the experimental conditions.

As such, the music memory task was designed so that every one of the 18 pieces of music was practiced as many times as needed for a participant to reach an ability to perfectly re-compose them from a forced choice design. The memory task was therefore inherently self-paced. In practice, due to the individual differences in music sensitivity and perceptive ability, some participants learned only some of the 18 music compositions with 100% accuracy on these re-composition retrieval/practice questions. Thus, for practical reasons, we put a 2-hour threshold on training day so that participants did not get trapped in the task forever. To facilitate analysis of the subsequent visual sequence learning data from day 2, we therefore marked the music sequences that were still re-composed with mistake(s) by the end of the encoding phase as ‘*unlearned’*.

#### Visual encoding phase (Day 2)

On the second day, prior to the Visual Encoding phase (see next section), the participant was first given a music memory recognition task for the 12 pieces of regular and irregular audio heard on the first day. We excluded isochronous clips because they did not contain any musical variations and shifting any notes could be easily detected. This task enabled us to identify "familiar learned" pieces that might nevertheless have been forgotten by subsequent day, giving finer grained information on when music memory influences other sequence learning (if at all). To test retention of each piece on day 2 in a reasonable time frame, we gave the participants a forced choice between 3 complete 8s audio samples and asked them to select the studied music composition from two lure versions of that sequence. To equalize the difficulty of recognition questions for each music, the lures were composed by shifting a few (1 to 3) notes in the temporal dimension (here we refer to rhythmic/accent timing patterns within a given tempo composition) from the original music or replacing it/them with notes belonging to the same chord within one octave away. The lures were very similar to the original clips such that participants needed good memory for day 1 music to answer the questions. The participant was tested on each Day 1 music (6 regular and 6 irregular) piece only once.

Once participants had been familiarized with the 18 pieces of music (and demonstrated varying degrees of retrieval and retention success as described above), the main Visual Encoding (learning) phase of the experiment began. Fig 3B shows the paradigm of this learning phase. During this phase, participants learned 36 distinct sequences of four novel abstract and irregular shapes. Each shape sequence was paired with one piece of 8-second music, making each shape correspond to 2 seconds of the music. The participant learned all 36 sequences in randomized order within a run, with five runs total (therefore, there were five learning repetitions per visual sequence). For each trial, the testing screen successively presented the four shapes in the correct order while the paired music was played. Then the participant saw a blank screen, holding the shapes in their mind, and listened to the music again, but played at a faster tempo of 6 seconds. We chose this duration of working memory gap based on participants’ feedback and performance during piloting. Right after this 6s break, the participant would repeat the sequence he/she just learned by clicking keyboard bottoms corresponding to the shapes. Once each shape/bottom was clicked, the corresponding 2 seconds of music to the selected shape would be played simultaneously. Participants could not change their selection even if they noticed that they made a mistake. The participant would see the feedback on whether he/she repeated correctly or not after this re-ordering practice. Critical for testing our predictions about music serving as a temporal template– 18 of the 36 visual sequences were paired with the 18 old music sequences, and the rest were paired with 18 novel new music compositions (with the same general properties). This enabled us to characterize arbitrary visual sequence learning curves in the *Encoding* phase as a function of having different levels of music familiarity as a backdrop and whether the music was *regular* or *irregular*.

#### Final retrieval phase (Day 2)

The experiment ended with a final retrieval phase that tested participants’ final memory of the new visual sequences and all the music sequences. In the visual sequence memory retrieval task, each participant was presented with visual sequence shapes and needed to re-order the shuffled shapes into the correct temporal order (the hallmark of a sequence). In each trial, the screen showed four shapes aligned in a row in shuffled order. Participants had 12 seconds to type in the corrected temporal order number for the shapes displayed. Without receiving feedback, they were allowed to change their answers within the 12s window. No music was played this time, providing a "pure" test of the final memory for the visual sequence structure. Each sequence was only tested once.

On the other hand, the musical sequence memory task was an error-detection task. In each trial, the participant first heard a version of one piece of music (8 seconds). Only *regular* and *irregular* music would be tested (24 in total). Because the control condition was monotonic sound lacking tonic harmony with steady temporal intervals, memory for the control condition was not tested (in simple terms, there was no opportunity for an error in this condition). There was a potential difference (error) in each second of the *regular* and *irregular* music played in this task compared to the original version. The difference could come from changes in both pitch and temporal interval. 22 out of 24 melodies contained errors in this task. The number of errors varied across music because 1) the goal was to test if the participants could not only detect the error but also identify correctness: some music had no error while others had up to 8 errors; and 2) participants would not find a pattern to be able to predict errors, instead of purely using their memory. The music would be played twice, back-to-back, for each test trial–because the participants needed to respond extremely fast once they detected an error, they would hear the music twice so that they could be prepared to respond after the first time’s detection. During the first repetition, the participants would not be asked to respond but to pay close attention to detect which second(s) of the piece contained error(s). During the second replay, the participants needed to click a button as soon as possible when the error occurred while the music was played. Each piece of music was tested once. The music memory task used a different probe from the re-ordering test for the visual sequence memory task because 1) ordering shuffled four parts of music might be easy due to humans’ comprehension sensitivity to music–for example, the syntax and learned schema for music that humans acquired over the course of lives could make putting a song in order too easy, and 2) this error detection task required faster reaction and can provide more precise measurement for music memory (score of 0 to 8 here).

### Statistical analyses

We conducted all data analyses and processing in R (https://cran.r-project.org). We used the *lme4* package [48] for generalized mixed-effect linear model implementation. Our main question of this study was how music with different levels of predictability affects concurrent visual sequence learning. Based on this goal, our analysis mainly focused on visual sequences retrieval memory performance and visual sequence encoding progress (learning curves/speed), examining these separately as a function of the experimenter-created music conditions described above.

#### Visual sequence encoding

Our task was designed such that during the encoding phase, because participants retrieved the visual sequences at the end of each trial, we could use their performance (either correct or incorrect per each trial) to derive learning rates. From this, we computed an index of such learning speed: at what learning stage/run each visual sequence was successfully learned (defined as the participant constantly correctly re-ordering the sequence in the subsequent runs after that learning stage). This was termed *the successful acquisition phase*, and for each run, we could then compute how many compositions had achieved this successfully-learned status. To test how fast participants acquired the visual sequences in each condition, we used a liner mixed effect model to quantify how music regularity and music familiarity together modulate the successful acquisition phase for each visual sequence (at which encoding stage was each sequence acquired), treating participants as random effect for the intercept.

#### Visual retrieval task

At the end of the experiment, visual sequential memory retrieval performance was evaluated by two measurements: the retrieval accuracy and response times for successful learned sequences (time used to re-order shuffled shapes), with faster correct-trial response times indicating a stronger memory strength for learned information. To be noted, because the response time analysis only looked at correct trials, we would not assume the patterns of the two measurements to be consistent all the time. For example, Condition D (*unlearned irregular*)’s retrieval accuracy showed no difference compared to control but had faster response time for correct trials than control. It might suggest that although participants did not encode more information overall in this condition, they had a stronger memory for what they successfully encoded. Thus, we hoped these two measurements together could provide more information about memory outcomes under different conditions. In order to test how music familiarity and regularity affected these two measures, we utilized mixed effect models to test how the two fixed effects and repeated measures (participant) as the random effect (to estimate individual-level intercept) predicted trial-by-trial retrieval performance (two separate models for retrieval accuracy and reaction time, see S1 and S3 Tables for model syntax). Because retrieval accuracy for each sequence was either correct or incorrect, we used a mixed-effect logistic regression model to test how the regressors affected retrieval accuracy. We used a Kenward-Roger approximation and the parametric bootstrap method for getting denominator degrees of freedom and p values for model regressors [49]. We used the same regressors on a mixed-effect linear model when testing how response time was affected by these factors and the interaction effect. After detecting significant interactive effects, we used the *lsmeans* package to calculate Tukey’s HSD post hoc test to compare pair-wise differences between conditions. Estimates of mean differences and confidence intervals were used to test effect sizes and can be found in S2 and S4 Tables.

## Results

### Manipulation checks

Due to the novelty of our task paradigm and task stimuli, we tested participants’ memory for all *Regular* and *Irregular* music stimuli we created at the end of the task (Day 2). This allowed us to verify that participants had better familiarity with "old" music that was shown on Day 1, and also that novel music was learned over the course of the second day. Fig 2A shows the music retrieval performance distribution. The average score on our music error detection task was 6.2 out of 8 for old music, and the new pieces had an average score of 4.8, significantly lower than old music (t(1222) = -3.917, p < .001). There were also significant differences between memory performance for *regular* and *irregular* pieces in both new and old conditions (new: t(609) = -3.824, p < .001, old: t(609) = -2.345, p = .009). This result was important because 1) better scores for old music validated our plan to compare novel shape sequence learning for two experimentally-created levels of music familiarity; and 2) the result that old *regular* music achieved better memory scores than old *irregular* music validated that our regularity manipulation was successful. However, because this study aimed to test regularity’s influence via a form of schema effect, this did create a need to equate the visual sequence learning conditions within a given level of our familiarity manipulation, such that they did not differ in background music familiarity but only in the intended perceptual/sequential structure differences of those compositions.

Participants’ music learning performance on the first day varied considerably. 25 out of 48 participants finished perfectly, learning all 18 pieces of “old” musical sequences (were able to re-compose the music by filling the five blanks perfectly–see *Methods*), although the majority of participants learned most of the pieces (minimum: 6 pieces perfectly learned, mean: 15.4/18). As noted above, we also found that participants learned significantly more *regular* music than *irregular* music to this perfect performance criterion (F(1, 609) = 12.06, p < .001, cohen’s d = 0.381). We explored whether individual training differences might affect such differences in music learning performance. Replicating prior observations [50], we found a positive correlation between music training history length and music learning performance on Day 1 (Pearson’s r(49) = .499, p < .001). Learning performance was defined as the ratio of perfectly learned pieces of music out of 18 minus total time used to finish the whole task, z-scored across participants. This measurement combined the proportion of music stimuli successfully learned and the time taken to learn them, such that higher scores reflected learning more stimuli in less time. The correlation between training history and learning performance aligned with past work suggesting music training can improve the ability to identify the structure of atonal(irregular) music [51].

Because participants learned more *regular* music perfectly during training, and participants with different music skills might end up with different levels of memory strength for Day 1 music, we adopted a strict definition of what music would be considered “old” for analysis, in order to avoid varying levels of familiarity among different pieces of music and participants providing an alternate explanation for *regular* vs. *irregular* condition differences. Specifically, we opted to analyze “old” music as the veridically predictable music (*learned* music) using a strict criterion in which music, whether *regular* or *irregular*, needed to be perfectly learned on Day 1 to be categorized as “*learned*” music in Day 2 analyses. This enabled us to maintain a factorial design in which we could compare how schematic *regular* and *irregular* “familiar/old” music affected visual memory vs. novel music. In this way, we ensured a strong learned vs. novel music comparison (we knew the participant could retrieve each detail of the piece in both regular and irregular learned music conditions), even though irregular music compositions were less frequently mastered to 100% (see Method).

When all the Day 1 music stimuli were tested again on the second day (excluding isochronous Control sequences–see *Methods*), the accuracy percentage ranged from 33.3% to 100% (mean 85.1%, standard deviation 16%). There was no significant difference between the accuracy of *regular* musical sequence versus *irregular* musical sequence across participants (repeated measures ANOVA: F(1,609) = 0.816, p = .367, η^2^_generalized_ = 0.0008), validating that the participants were able to recognize the majority of the music even after a day of learning, across both levels of regularity.

In summary, due to potential individual difference in music learning, we labeled musical sequences as ‘*learned’* stimuli if the participants successfully acquired that piece on the first day and successfully recognized it on the second day. This strict criterion enabled us to control variable and test the hypothesis that familiar music’s predictable structure could affect new memory formation. All the unsuccessfully-learned music pieces from Day 1 and all 18 remaining sequences that were not present on the first day were grouped into ‘*unlearned*’ music–providing a stringent test of our known vs. less familiar comparison (Note: our pattern of results was qualitatively unchanged when “*unlearned*” excluded trials with music that was experienced on Day 1 but not successfully learned; see S7–S9 Tables and compare to S1–S4 Tables. Control (monotonic) music that appeared on Day 1 (6 clips in total) were all marked as ’*learned’* and the rest were marked as ‘*unlearned’*. This was for easier visualization when comparing with other music conditions so we could do a 2x3 factorial linear regression analysis (even though the Control tone sequences could not be “learned” per se).

### Visual sequential encoding: How did music affect the learning curves for parallel information?

**Fig 4:**
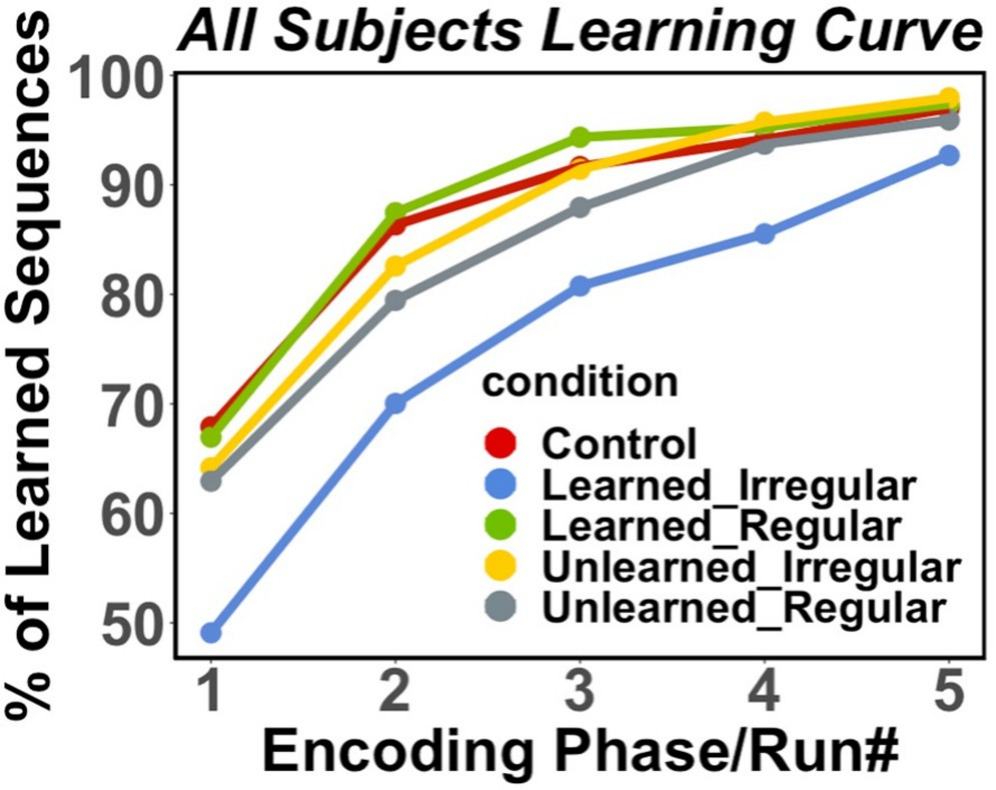
Visual encoding phase. This figure represented the cumulative learning curve during visual encoding. The plot illustrates the average proportion of visual sequences learned so far at each phase/run as a function of each music condition. The significantly slowest learning happened in the *learned-irregular* condition. To simplify the visualization, we combined *learned control* with *unlearned control* condition performance for each phase because 1) there were no statistically significant differences between them (Tukey’s HSD: p = .9113) plus 2) conceptually, the monotonic sequences, without dynamic changes in notes and temporal intervals, could not be “learned” and schematized thus were identical in both familiar and unfamiliar conditions.

Having the participants practice sequence retrieval during each trial of the visual encoding phase enabled the investigation of visual sequence learning qualities such as learning speed as a function of the music conditions. We computed the *successful acquisition phase* (the run number between 0–5 runs) separately for each visual sequence (see *Methods*). This value represented when the participant had learned each sequence. The cumulative acquired sequence proportion during each run of encoding for each condition (e.g., *learned-irregular*) provided insight into the visual sequence learning rate. We ran mixed effect linear model on participants’ sequence acquisition learning rates to test whether how fast participants learned was affected by music familiarity and regularity. We found significant effects of music regularity (regularity: Estimates = 0.53, 95% CI [0.31,0.75], df = 1682.9; Type III Wald Chi-square Test: χ²(2) = 30.238, p < .001). We also found a highly significant interaction (Estimates = -0.4, 95% CI [-0.68, -0.11], df = 1685.1; Type III Wald Chi-square Test: χ²(2) = 19.4, p < .001). Fig 4 shows the visual sequence learning curve for each condition. Pair-wise comparisons unpacking these fixed effects indicated that *learned*-*irregular* music-paired shape sequences were learned the slowest–beginning and maintaining a significant deficit from the other conditions until the fifth encoding phase (See S6 Table for all comparisons p values and effect sizes).

### How well did participants retrieve the visual sequences after learning? Visual memory retrieval test

**Fig 5:**
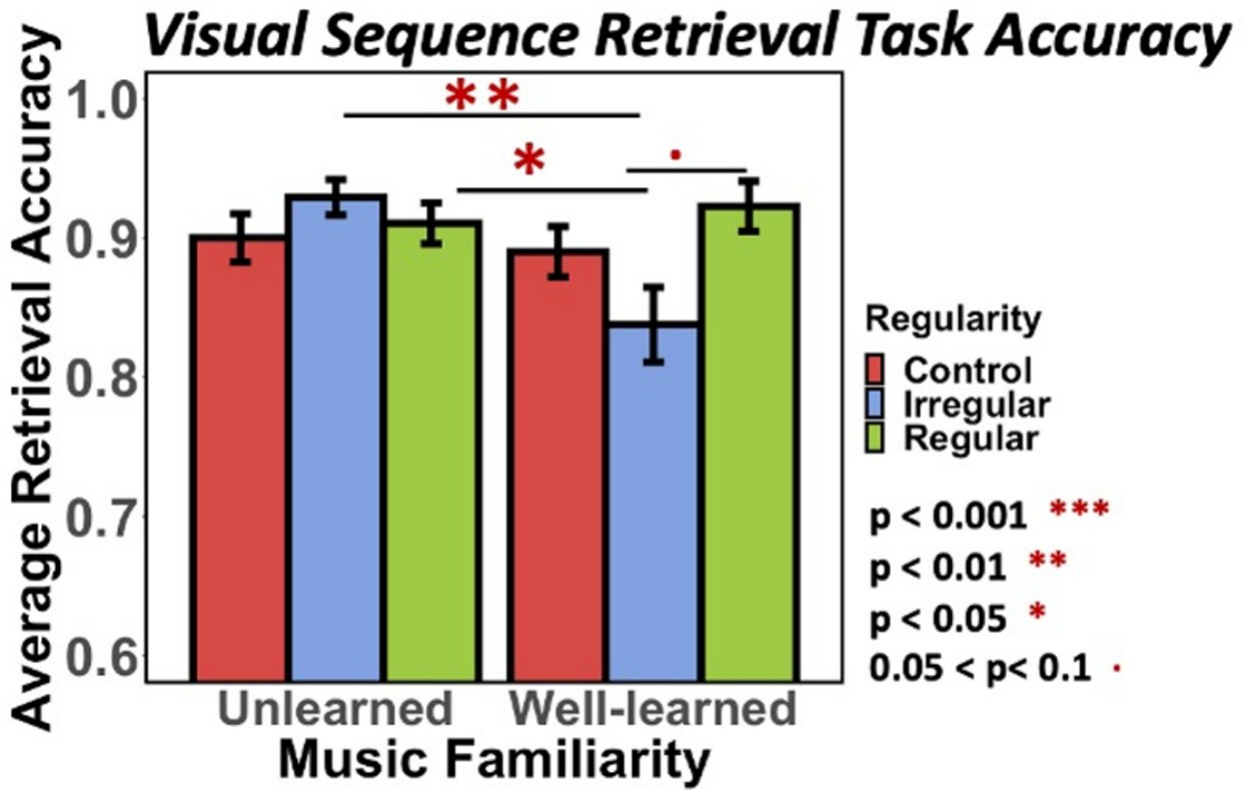
Visual sequence retrieval performance. The bar plot represents average retrieval accuracy for visual sequences comparing *learned* vs. *unlearned*, three regularity conditions. Average retrieval accuracy for each condition from left to right is: 0.901, 0.93, 0.917, 0.888, 0.84, 0.926. Standard deviations from left to right are: 0.299, 0.255, 0.277, 0.316, 0.357, 0.27. Tukey’s HSD pair-wise comparisons indicated that the most difference came from the *learned-irregular* group.

By Day 2, participants scored either correct or incorrect for each trial during the final visual sequence retrieval task. Overall final visual task accuracy percentage across participants ranged from 58.3% to 100% and 90% on average. To test whether final retrieval performance differed across the preceding music-encoding conditions, we used a mixed-effect logistic regression model and asked how trial-by-trial retrieval accuracy was affected by music familiarity and regularity condition (fixed effects), with individual participants as a random effect. Results showed a significant interaction between music familiarity and regularity (See S1 Table for model details: odds ratio = 2.65, 95% CI [1.19,5.92]; Types II Wald Chi-square Test: χ²(2)_Familiarity x Regularity_ = 7.73, p = .02). Looking into the interaction effect, Tukey’s pair-wise comparisons showed that the *learned-irregular* music condition resulted in lower visual retrieval accuracy than most of the other music conditions (Fig 5: *learned-irregular < learned-irregular*: p = .007, mean difference = 0.09, 95% CI [0.02, 0.16]; *learned-irregular* < *unlearned-regular*: p = .043, mean difference = 0.08, 95% CI [0.00, 0.15]; *learned-irregular* < *learned-regular* : p = .063, mean difference = 0.08, 95% CI [0.00, 0.16]; see S2 Table all pairwise comparisons), indicating a disruptive effect of this type of music on visual sequences encoding whereas other music conditions showed trending improvement or no changes compared to control. See S5 Table for average retrieval accuracies and standard deviations for all conditions. Notably, learning impairment was absent from *unlearned irregular*, which oppositely showed an improving trend, indicating the disruptive effect was tied to music memory.

**Fig 6:**
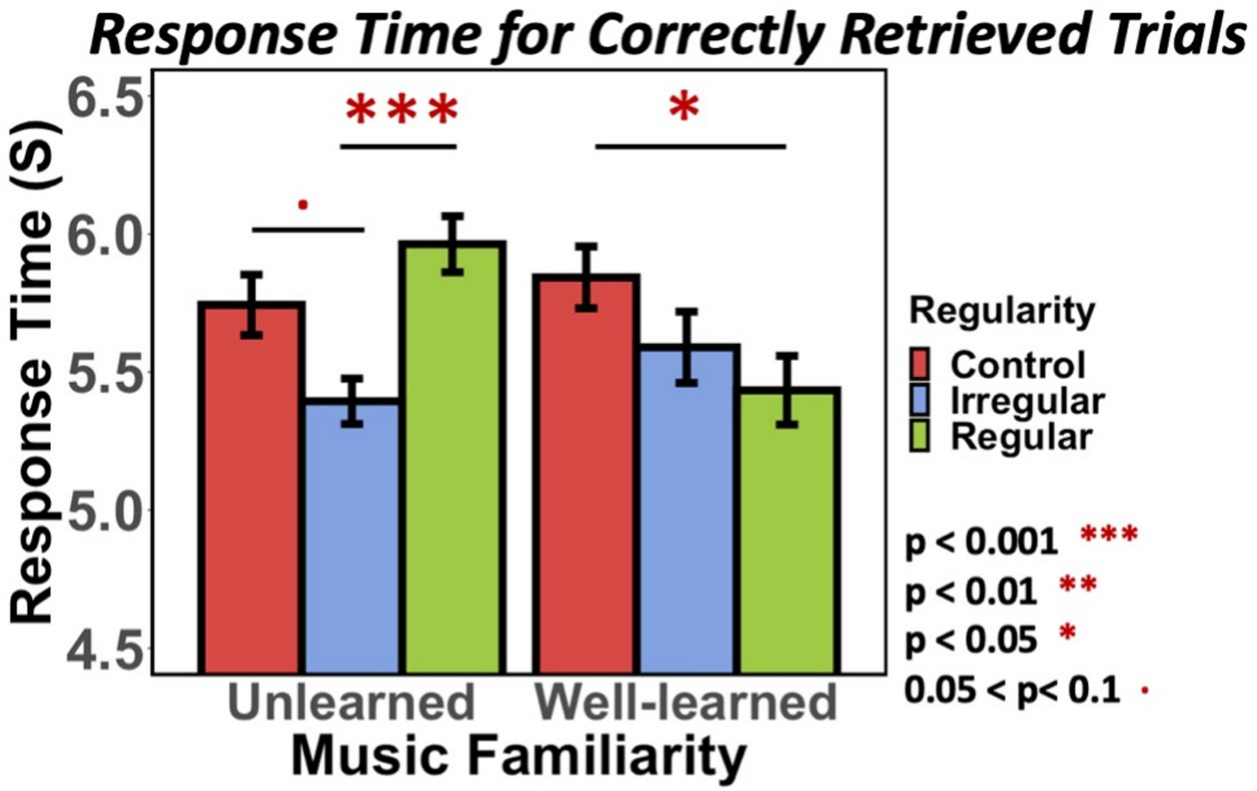
Visual retrieval task–response time for correctly trials only. The bar plot represents average response times for correctly retrieved trials only for visual sequences comparing *learned* vs. *unlearned*, three regularity conditions. Average reaction time for each condition from left to right is: 5.74, 5.33, 5.9, 5.8, 5.57, 5.41. Standard deviations from left to right are: 2.05, 1.83, 2.06, 2.14, 1.95, 1.9.

While retrieval accuracy provided evidence for a detrimental effect from learned-irregular music, we also examined response time (RT) differences for correctly retrieved trials among conditions. Many studies have shown that RT in memory retrieval tasks can help represent the strength of memory [52–54], especially for correct trials–with a faster reaction or response in successful retrieval, implying a better memory and higher confidence. In other words, if an individual responds correctly on object sequencing for two music conditions but is twice as slow for one music condition than the other, this additional RT information is necessary to reveal evidence that a successful retrieval event was nevertheless more effortful for that condition. Using a linear mixed-effect regression model, we found a significant effect of regularity (See S3 Table for model details: Estimates = 0.33, 95% CI = [-0.61,-0.06]; Type II Wald Chi-square Test: χ²(2)_Regularity_ = 5.58, p = .05) and an interaction between regularity and music familiarity ((Estimates = 0.48, 95% CI = [0.11,0.84]; Type II Wald Chi-square Test: χ²(2)_Familiarity x Regularity_ = 18.44, p < .001). To understand this interaction, comparing the simple effects between pairs, interestingly, we found that within the *unlearned* condition, the *irregular* conditions had significantly faster response times (Fig 6: *unlearned-irregular* RT < control RT: p = .06, mean difference = 0.41, 95% CI [-0.03 0.84], *unlearned-irregular* RT < *unlearned-regular* RT: p < .001, mean difference = 0.6, 95% CI [0.18 1.00], see S4 Table for estimated mean differences and 95% confidence intervals) in addition to its high accuracy. Conversely, and supporting one of our hypotheses for this study, for visual sequences encoded with *learned* musical sequences, the *regular* condition had a significantly faster reaction time than the control (*learned-regular* RT < control RT: p = .013, mean difference = 0.59, 95% CI [0.08 1.10]).

Together, the final visual sequential retrieval task provided two insights: (1) overall retrieval accuracy suggested that when visual sequences were encoded with *learned* music stimuli, their subsequent sequence retrieval accuracy was disrupted by *learned-irregular* music at encoding. (2) RTs for correct trials (an indicator of the strength of learned sequence associations) significantly benefitted from *learned-regular* music (as well as, interestingly, *unlearned-irregular* music).

## Discussion

In broad terms, this study aimed to investigate how a specific music feature–music predictability–modulates concurrent learning of information from other modalities. Inspired by memory theories that pre-existing knowledge can impact how easily people learn associated new information [15, 55], we tested whether predictable music would be more helpful to parallel learning than less predictable music given that it provides a clearer temporal context trace for new encoding (e.g., as in language, knowing what grammatical feature or note is coming or likely to come next). Our results suggested that music “familiarity” and “regularity”, also known as veridical and schematic knowledge in music literature [27], as operationalized in our study, interactively affected the learning performance of visual sequential information (as evidenced by the significant interaction effect from the mixed-effect linear model). In summary, we found consistent detrimental effects of the *learned irregular* music (Condition B) on visual sequential learning: participants learned the abstract shape sequences the slowest and with the least accuracy in this condition during visual sequence encoding. Similar patterns were also reflected in the final visual sequence retrieval accuracy analysis. On the other hand, the visual retrieval task performance revealed a beneficial effect of concurrent music that was regular in structure and already known (Condition A). In fact, for shape sequences that were successfully learned, participants retrieved them faster in subsequent tests if they were learned with *learned regular* music. Together, these outcomes from the familiar music conditions supported our prediction of benefits of predictable music structure and detriments of irregular structure on learning. Interestingly, however, we also observed a surprise outcome for our theoretical framework: in the final retrieval task, visual sequences that were encoded with learned regular music or alternatively with the unlearned irregular music (the least predictable condition) showed the highest accuracy and decreased retrieval reaction times. This differed from our original hypothesis, because it demonstrates something akin to a U-shaped relationship between predictability level and visual sequence. Although interpreting the benefit of unlearned irregular music post-hoc is challenging, we speculate that this may relate to arousal modulation and working memory improvements, as suggested in some music literature [35, 56] (we elaborate on this idea more below).

As summarized above, congruent to our hypothesis, we found evidence for strong opposite effect of *learned regular* music versus *learned irregular* music. This might support the importance of a concept from music science that the hierarchy of music structure is an important factor for it to serve as a schema that will affect concurrent learning (but see continued discussion of the *unlearned irregular* outcome below). Following syntactical rules allows music to have hierarchical or high-level relationships between music elements–a major trait behind what makes music “musical” and analogous to language [57, 58]. Indeed, outside of music, a study found that hierarchical stimulus structure might benefit learning new associative information by strengthening and clustering the relationships between items [59]. Together with support from schema theory, which has highlighted how stable prior knowledge could benefit associated information learning [15, 17], we postulated that *learned regular* music (Fig 1: Condition **A**) provides a structured temporal hierarchy where upcoming elements and timing are known and predictable, which could be a beneficial sequencing context for associated visual item sequencing. A recent study provided evidence for this idea by showing cross-modal aid from familiar motor sequences on parallel visual sequence learning [60]. In addition to the observed higher average retrieval accuracy for visual sequences paired with *learned regular* music compared to *learned irregular* music, we found evidence that *learned regular* music also strengthened sequence memories that formed: sequences that were correctly retrieved were retrieved the fastest when they had been previously learned with this paired music as a mnemonic.

An important feature of our design is that the monotonic control (as opposed to silence) allowed our results to determine if such beneficial effects only come from having a regular temporal presentation, per se, which is known in some designs to guide attention and improve visual memory encoding [23]. Since our control condition consisted of isochronous streams of tone which hold such a regular rhythm (also associated with improving visual processing [61]), we argue that attention modulation from simply having a rhythm present is not the only mechanism behind the music effects on memory observed–rather the benefit is likely attributable to the utility of the music as a supporting contextual cue for what comes next in sequence. Our surprising beneficial effects found from *unlearned irregular* music, also speak against the presence of a simple rhythm in the control being optimal for learning.

Additionally, in line with our theoretical framework, we found detrimental effects of the *learned irregular* music (Fig 1: Condition **B**), which might be due to the destruction of hierarchy and its conflict with our memory, resulting in attentional diversion. Prior studies have found that memory-governed (veridical expectation) and rule-governed (schematic) knowledge influence music processing and music priming independently [19, 62]. Moreover, it has been suggested that listeners depended more on the rule-governed syntactical prediction during music processing since both behavioral and neuronal data showed that knowing the upcoming event (veridical knowledge) could not prevent ‘error detection’ feeling during processing syntactic-violated music [18, 20]. In our design, by stripping away the hierarchy and syntactic characteristics of music in the *irregular* condition [63, 64], those stimuli were designed to fail to produce higher-level syntactic meaning. Our stimulus validation using MIR toolbox provided quantitative evidence for a looser temporal structure for our irregular music. This fragmentation of music’s language is known to result in neural “prediction error”-related signals in, e.g., the N400 and MMN (mismatch negativity) [29, 30, 65, 66]. Based on these prior findings, one would predict a hindering effect of *learned-irregular* music on parallel visual encoding, reflecting memory for the composition predicting music schema violations, eliciting strong conflict between the specific composition’s memory and “correct” flow of the structure according to schematic music memory, perhaps drawing mnemonic resources towards the “oddity” in the composition that elicited strong prediction errors during its learning. As such, our *learned-irregular* music was designed to fail to provide as stable or consistent a temporal sequence template for new learning. There are related mechanism possibilities along this line, which include proactive interference effects and divided attention effects. For example, some studies have suggested that auditory experiences with mixed familiarity and novelty (déjà entendu: the auditory analog to déjà vu) are attention-grabbing and might interfere with the working memory capacity [67, 68]. In our scenario, the *learned irregular* music, although familiar, was structured in a novel pattern/grammar from the broader music syntax we know, and thus might pull mental attention toward a metacognitive state and away from the visual task and possibly disrupt working memory allocation for the visual sequencing to be performed. Indeed, knowing this syntactic violation is coming (the fact this manifest in the well-learned/familiar condition) may contribute to its proactive interfering effect on concurrent learning processes. In real-world settings, this type of situation may most strongly manifest in cases where a listener is processing music they have heard before, but from a fundamentally poorly-known music style for them (such as music from other cultures).

As noted above, however, our results from the *unlearned irregular* condition suggested that syntactical expectation or the hierarchical structure alone was insufficient to benefit or detriment cross-modal sequential learning. We observed benefits from music that either has or lacks both sources of predictability (familiarity and regularity), while detriments were from music conditions that lacked one form of predictability but not the other. Though *unlearned regular* (Fig 1: Condition **C**) music did not reveal a significant detrimental effect relative to Control, the group-level results also showed that listening to this type of music did not benefit visual sequential learning differently than the control condition. In fact, response time results suggested a subtler detriment of *unlearned-regular* music, with longer reaction times when retrieving a sequence correctly. This firstly might add evidence to prior studies using unfamiliar (*regular*) music, which have shown consistent results with our observations: that *unlearned-regular* music was associated with the qualitatively weaker learning outcomes [12, 69, 70]. One interpretation we have is that when there is a conflict between music veridical expectation and schematic expectation, this might counteract the ability of music to transfer informative temporal sequential information to cross-modal learning.

The surprising dimension of these outcomes was the fact that we found music lacking *both* familiarity and regularity (Fig 1: Condition **D–***unlearned irregular*) might actually strengthen parallel visual sequential memory. Due to the behavioral nature of this task, we have limited insight into the mechanism behind this observation. There is currently a lack of prior studies that used truly novel and irregular music as a stimulus during a memory encoding task. However, one intriguing possibility is that encountering small prediction errors (bearing in mind that our *irregular* music stimuli retained some tonal relationships, and were still subjectively “musical” to listen to on-the-whole) could increase physiological arousal level [71] to a moderate degree, which might instead improve memory performance. There is evidence for this benefit of moderate arousal in both music and non-music literatures [35, 69]. Such an application of Yerkes–Dodson law [72] is broadly consistent with decades of cognitive psychology work highlighting U-shaped relationships between arousal levels and/or difficulty of background processing and how cognitive resources are allocated to the task in the foreground (from language processing to spatial sequencing tasks [73, 74]). In exploratory follow-up performance cluster analysis (S1 Text), we found evidence that people with more music training might benefit more from this type of music. We postulate that when people gain better understanding of the structure of irregular music, this type of music would become less unpleasant. A study suggested surprise during music listening, while the music has moderate level of syntactical predictability can give rise to pleasure [75]. In this way, novel (unlearned) less-regular music might promote attention and learning during a cross-modal cognition task via arousal mechanisms, whereas when the music becomes familiar and people gain veridical expectations of it when listening, this discordant structure would begin to instead occupy attention and generate more disruptive effects. While future research is need to elucidate the mechanism, one compelling insight from our beneficial effects from *unlearned-irregular* music is that there may be potential clinical uses of such types of music as arousal and stress modulators to improve memory [69, 76, 77].

In summary, in this study, we put forth a hypothesis that using music during episodic memory-like learning could modulate the new sequence memory strength and learning speed via the “schema” framework: the syntax and temporal structure from music memories can provide a template for facilitating temporally-ordered memory of new, paired stimulus associations. Our study synchronized differing degrees of predictably-structured music with abstract shape sequences, creating an interactive learning environment that was found to modulate visual learning performance. It is worth noting, in an exploratory analysis (S10 Table), we compared the impact of music heard on Day 1 (old) versus new, never-heard-before music on final visual memory performance, irrespective of if the music met our strict “learned” criteria. We found a qualitatively similar pattern of familiarity (now merely “prior exposure” to the music) and regularity on final retrieval accuracy, and a significant familiarity*regularity interaction when examining response time. However, significance was attenuated (e.g., no familiarity*regularity interaction in accuracy). We take this as support for our view from the primary significant interaction results showing that memory for the compositions is a factor acting on the mechanisms through which regularity influences learning. In this alternative way of binning the data, now some ‘old’ music in the regular and irregular conditions exhibited weaker memory than others. This idea could be formally tested in future studies, which could manipulate the levels of prior exposure to music to disentangle whether an accurate memory trace is present (our current focus) from different degrees of familiarity (which might occur across both perfectly and imperfectly remembered compositions). This would further identify how different levels of music knowledge affect learning.

Although the present study approached the hypothesis from a cognitive perspective, there exists extensive behavioral and neuroscience support for the schema theory we draw upon, and such data suggest that old memories constantly interact with new information encoding and could potentially improve the new learning in a way that is not limited to a single modality [15, 16, 25, 60, 78–80]. Animal studies have shown that in the hippocampus, an area strongly associated with memory encoding and consolidation, schemas and associative memory were represented by hierarchically-organized neural representations, suggesting that relevant memories share overlapping neural states even when the component pieces of associative information were of different types (e.g., item and spatial location) [6]. In such ways, the music that is ubiquitous in our lives could potentially modulate our long-term memory for episodes, study materials, and beyond in complex ways ‐ and our present study put some of these ideas to the test. Here, we found a strong interaction effect between music familiarity and regularity on parallel visual sequence memory encoding, where music could be beneficial to new learning when it featured both familiarity and structural regularity (surprisingly, we also found evidence for such benefits if it exhibited neither of these traits). Moreover, music significantly disrupted new sequence learning in scenarios where music was well-learned but lacked regularity (suggesting an overall U-shaped relationship between our manipulated drivers of predictability and memory). On the individual differences level, our study suggests that the pattern in how different types of music influence sequence learning could be shaped by whether people have more or less music training. The perspectives offered by our pattern of results might help provide an explanation for varied results in past literature testing music’s effect on various types of memory–the individual music listener’s traits, and the properties of the music itself might significantly shape our memory outcomes.

Importantly, in our design, music was not merely played in the background, but its components were paired with the new stimuli to be learned. Therefore, it does not answer the questions such as ‘does listening to music in the background during study help learning?’. However, it demonstrates a framework for understanding and designing music as a mnemonic device–insights helpful for future applications of music in both educational setting and clinical setting.

The current study also had several limitations to be followed-up on in future work. The first limitation lies on the high retrieval performance across conditions, suggesting potential ceiling effects. In a follow-up MRI study (in preparation), we decreased the encoding time to avoid this potential ceiling effect. Moreover, the current behavioral data present limited evidence for how exactly each type of music affected learning. For example, why is *unlearned irregular* music beneficial, and does it influence visual learning via a different mechanism (as we speculated) compared to *learned regular* music? We hope neuroimaging methods in follow-up studies reveal neural mechanisms for how different music affects learning.

Another limitation relates to generalizability of these findings to dynamic real-world scenarios involving music (tied largely to our highly-controlled laboratory design and novel lab-based stimuli). One remaining question related to this limitation is how the music exposure studied here might influence learning relative to silence, rather than the monotonic control sounds we used. It is worth noting that during initial piloting, we ran an identical version of the final task (N = 21) using a silence control condition in place of the monotonic control, and we observed a similar pattern of results, with *familiar regular* music again showing benefits (in this case, relative to silence). However, we acknowledge this pilot sample was relatively small, and thus further studies providing higher-powered direct comparisons against a silent baseline are warranted, especially for translational and applied purposes (given that silence is a natural reference point in the real world). Such work could also shed light on the benefit we observed with *unlearned irregular* music over monotonic controls. Specifically, do these unfamiliar and less predictable compositions upregulate attention as we speculated, or are they effectively ignored given their lack of predictability, akin to silence? While we stress that the control condition we employed is strict and helps limit some alternative interpretations when comparing our music conditions, larger-scale experiments systematically pitting music conditions against silence would help adjudicate these possibilities.

Also, although our results point to the importance of music temporal expectancy for parallel learning processes, we cannot exclude the possibility that other music features might be involved in daily learning outcomes (e.g., emotionality/valence). Whereas this study created irregular music by violating the structural and temporal relationship between notes by shuffling the notes of regular music, future studies can also look at how violations in music key relationships modulate parallel learning. Another consideration is that here we used brief music compositions that followed the same music style and with the same tempo. While features like the duration of the compositions and the tempo were arrived at through various feasibility considerations during piloting, it is possible that our results might change given, e.g., a faster tempo (or a different timing for when the visual stimuli co-occurred with the music). We also note that we manually created abstract shapes to achieve novelty ‐ however, we did not formally manipulate or assess the structural similarity of the shapes (morphology could influence their memorability). Future tasks using these stimuli may consider including such measurements to systematically assess whether music is, for example, more beneficial for remembering relationships between shapes which are more difficult to associate with one-another. Although the scope of all such possibilities could not feasibly be addressed within a single study, we are excited to see this further explored in the field in future studies.

Turning outward from the intricacies of our present design, because sequential memory is an important component of episodic memory–in which humans typically combine separate modalities of information (sights, sounds, emotions) in sequence using a memory system optimized for forming huge networks and associations between events [81–83]–one prediction from our current study is that our ability to bind other elements of episodic memories together in time may be influenced by the varying properties of music that we manipulated. In the big picture, we might speculate from our findings that listening to music–and perhaps musical training (which strengthens our ability to process its structure) ‐ may impact how our memory systems encode and organize other experiences around us in our daily lives. As such the new insights from our study strongly motivate future studies into how we might use music as a potential memory aid.

## Supporting information

S1 Table Logistic regression mixed-effects model result for final visual retrieval accuracy. (PDF)

S2 Table Post-hoc Tukey HSD test on visual retrieval accuracy. (PDF)

S3 Table Logistic regression mixed-effects model result for response time. (PDF)

S4 Table Post-hoc Tukey HSD test on retrieval RT. (PDF)

S5 Table Visual retrieval performance summary. (PDF)

S6 Table Pairwise comparison: amount of learned sequences across encoding phase. (PDF)

S7 Table Model results excluding day 1 unlearned trials. (PDF)

S8 Table Post-hoc Tukey HSD test (accuracy) excluding old-unlearned trials. (PDF)

S9 Table Post-hoc Tukey HSD test (RT) excluding old-unlearned trials. (PDF)

S10 Table Model results comparing NEW (day2) vs OLD (day1) music. (PDF)

S1 Fig Day 1 training–music re-composition task. (PDF)

S1 Text Clustering analysis. (PDF)
