# Supplementary material for: Visual sequence encoding is modulated by music schematic structure and familiarity
Source: PLoS One. 2024 Aug 7;19(8):e0306271. doi: 10.1371/journal.pone.0306271 (PMC11305557; doi:10.1371/journal.pone.0306271)
Supplement: S1 Table — (PDF) [file pone.0306271.s001.pdf]

S1 Table

	accuracy		
Predictors	*Odds Ratios*	*CI*	*p*
(Intercept)	14.4	8.13 -25.49	**<0.001**
Familiarity [unlearned]	1.18	0.68 - 2.03	0.556
Regularity [Irregular]	0.58	0.33- 1.03	0.064
Regularity [Regular]	1.4	0.74-2.64	0.297
Familiarity [unlearned] * Regularity [Irregular]	2.65	1.19- 5.92	**0.017**
Familiarity [unlearned] * Regularity [Regular]	0.93	0.40-2.15	0.864
**Random Effects**			
σ^2	3.29		
$\tau_{00\ subID}$	1.75		
ICC	0.35		
N_{subID}	48		
Observations	1764		
Marginal R^2 / Conditional R^2	0.022 / 0.362		

Logistic Regression Mixed-effects Model Result: trial by trial retrieval accuracy predicted by music familiarity and music regularity with subject as random effect.

Model syntax: *glmer(accuracy ~ Familiarity*Regularity + (1|subID), data, family="binomial"(link = "logit"),control=glmerControl(optimizer="bobyqa",optCtrl=list(maxfun=2e5)))*
