# Supplementary material for: Visual sequence encoding is modulated by music schematic structure and familiarity
Source: PLoS One. 2024 Aug 7;19(8):e0306271. doi: 10.1371/journal.pone.0306271 (PMC11305557; doi:10.1371/journal.pone.0306271)
Supplement: S2 Table — (PDF) [file pone.0306271.s002.pdf]

S2 Table Post-hoc Tukey HSD test on visual retrieval accuracy

			95% Confidence Interval		
	**Comparison**	**Mean Difference**	**Lower**	**Upper**	**p adj**
**	Irregular_Unlearned - Irregular_Learned	0.0896	0.0157	0.1635	0.0073
	Control_Learned - Irregular_Learned	0.0473	-0.0307	0.1253	0.5115
	Control_Unlearned - Irregular_Learned	0.0609	-0.0171	0.139	0.2252
.	Regular_Learned - Irregular_Learned	0.0809	-0.0025	0.1642	0.0631
*	Regular_Unlearned - Irregular_Learned	0.0762	0.0015	0.151	0.0426
	Control_Learned - Irregular_Unlearned	-0.0422	-0.1064	0.0219	0.4162
	Control_Unlearned-Irregular_Unlearned	-0.0286	-0.0928	0.0355	0.7998
	Regular_Learned - Irregular_Unlearned	-0.0087	-0.0792	0.0618	0.9993
	Regular_Unlearned - Irregular_Unlearned	-0.0133	-0.0735	0.0468	0.9886
	Control_Unlearned - Control_Learned	0.0136	-0.0553	0.0825	0.9933
	Regular_Learned - Control_Learned	0.0335	-0.0413	0.1084	0.7971
	Regular_Unlearned - Control_Learned	0.0289	-0.0363	0.0941	0.804
	Regular_Learned - Control_Unlearned	0.0199	-0.0549	0.0948	0.9741
	Regular_Unlearned - Control_Unlearned	0.0153	-0.0499	0.0805	0.9852
	Regular_Unlearned - Regular_Learned	-0.0046	-0.0761	0.0668	1

The interactive effect between music regularity and level of familiarity on visual sequence retrieval accuracy was found to be significant. Thus, post-hoc pairwise comparison was conducted on all possible pairs of conditions. The table showed all statistical results from the Tukey HSD test comparing the mean retrieval accuracy between each pair of conditions. P value was adjusted and all significant pairs were highlighted. **($p<0.001$: ***, $p<0.01$: **, $p<0.05$: *, $0.05< p<0.1$: ·)**
