# Supplementary material for: Visual sequence encoding is modulated by music schematic structure and familiarity
Source: PLoS One. 2024 Aug 7;19(8):e0306271. doi: 10.1371/journal.pone.0306271 (PMC11305557; doi:10.1371/journal.pone.0306271)
Supplement: S3 Table — (PDF) [file pone.0306271.s003.pdf]

S3 Table

	ResponseTime			
Predictors	*Estimates*	*CI*	*p*	*df*
(Intercept)	5.9	5.58 - 6.22	**<0.001**	88.61
Familiarity [unlearnedl	-0.09	-0.35 - 0.17	0.488	1544.2
Regularity [Irregular]	-0.09	-0.39 - 0.21	0.554	1548.61
Regularity [Regular)	-0.33	-0.61 - -0.06	**0.018**	1545.93
Familiarity [unlearnedl * Regularity [Irregular]	-0.36	-0.74 - 0.03	0.067	1549.48
Familiarity [unlearnedl * Regularity [Regular)	0.48	0.11 - 0.84	**0.011**	1547.05
**Random Effects**				
σ^2	2.22			
$t_{oo\ sublD}$	0.84			
ICC	0.27			
N_{sublD}	48			
Observations	1596			
Marginal R^2 / Conditional R^2	0.017 / 0.287			

Linear Mixed-effects Model Result: trial by trial response time (excluding incorrect trials) predicted by music familiarity and music regularity with subject as random effect.

Model syntax: *lmer(ResponseTime~Familiarity*Regularity + (1|subID), data)*
