# Supplementary material for: Visual sequence encoding is modulated by music schematic structure and familiarity
Source: PLoS One. 2024 Aug 7;19(8):e0306271. doi: 10.1371/journal.pone.0306271 (PMC11305557; doi:10.1371/journal.pone.0306271)
Supplement: S4 Table — (PDF) [file pone.0306271.s004.pdf]

S4 Table Post-hoc Tukey HSD test on retrieval RT

			95% Confidence Interval		
	Comparison	Mean Difference	Lower	Upper	p adj
	Irregular_Unlearned - Irregular_Learned	-0.3844	-0.8866	0.1179	0.246
	Control_Learned - Irregular_Learned	0.2298	-0.3006	0.7602	0.8192
	Control_Unlearned - Irregular_Learned	0.0231	-0.5073	0.5535	1
	Regular_Learned - Irregular_Learned	-0.356	-0.9226	0.2105	0.4706
	Regular_Unlearned - Irregular_Learned	0.2038	-0.3045	0.7121	0.8629
***	Control_Learned - Irregular_Unlearned	0.6141	0.1778	1.0505	0.0009
.	Control_Unlearned-Irregular_Unlearned	0.4075	-0.0289	0.8438	0.0631
	Regular_Learned - Irregular_Unlearned	0.0283	-0.4513	0.5079	1
***	Regular_Unlearned - Irregular_Unlearned	0.5882	0.179	0.9973	0.0006
	Control_Unlearned - Control_Learned	-0.2067	-0.6751	0.2618	0.8075
*	Regular_Learned - Control_Learned	-0.5858	-1.0948	-0.0768	0.0134
	Regular_Unlearned - Control_Learned	-0.026	-0.4692	0.4172	1
	Regular_Learned - Control_Unlearned	-0.3791	-0.8881	0.1299	0.2748
	Regular_Unlearned - Control_Unlearned	0.1807	-0.2625	0.6239	0.8544
*	Regular_Unlearned - Regular_Learned	0.5598	0.0739	1.0457	0.0132

The interactive effect between music regularity and level of familiarity on response time of correctly retrieved sequences was found to be significant. Thus, post-hoc pairwise comparison was conducted on all possible pairs of conditions. The table showed all statistical results from the Tukey HSD test comparing the mean retrieval accuracy between each pair of conditions. P value was adjusted and all significant pairs were highlighted. **(p<0.001: ***, p<0.01: **, p <0.05: *, 0.05< p<0.1: ·)**
