# Supplementary material for: Visual sequence encoding is modulated by music schematic structure and familiarity
Source: PLoS One. 2024 Aug 7;19(8):e0306271. doi: 10.1371/journal.pone.0306271 (PMC11305557; doi:10.1371/journal.pone.0306271)
Supplement: S5 Table — (PDF) [file pone.0306271.s005.pdf]

S5 Table Visual Retrieval Performance

Condition	Mean	Standard Deviation
**Retrieval Accuracy**		
**Overall**	0.9	0.293
**Learned Regular**	0.926	0.27
**Unlearned Regular**	0.917	0.277
**Learned Irregular**	0.84	0.357
**Unlearned Irregular**	0.93	0.255
**Control**	0.894	0.307
**Reaction Time for Correct Trials (s)**		
**Overall**	5.67	1.75
**Learned Regular**	5.41	1.9
**Unlearned Regular**	5.9	2.06
**Learned Irregular**	5.57	1.95
**Unlearned Irregular**	5.33	1.83
**Control**	5.79	1.79
