# Supplementary material for: Visual sequence encoding is modulated by music schematic structure and familiarity
Source: PLoS One. 2024 Aug 7;19(8):e0306271. doi: 10.1371/journal.pone.0306271 (PMC11305557; doi:10.1371/journal.pone.0306271)
Supplement: S6 Table — (PDF) [file pone.0306271.s006.pdf]

S6 Table Pairwise comparison between conditions on amount of acquired sequences during each run of encoding phase (all subjects)

Run# of encoding	**p value of T-test between/effect size (cohen's d)**	*Control*	*Learned_Irregular*	*Learned_Regular*	*Unlearned_Irregular*
1	Learned_Irregular	7.910E-05/0.63			
	Learned_Regular	8.372E-01	1.110E-03/0.584		
	Unlearned_Irregular	4.160E-01	5.537E-03/0.495	6.013E-01	
	Unlearned_Regular	2.796E-01	1.078E-02/0.462	4.511E-01	8.161E-01
2	Learned_Irregular	3.550E-05/0.63			
	Learned_Regular	7.704E-01	1.254E-04/0.63		
	Unlearned_Irregular	3.227E-01	5.195E-03/0.478	2.694E-01	
	Unlearned_Regular	7.017E-02/0.37	3.551E-02/0.344	6.996E-02/0.382	4.744E-01
3	Learned_Irregular	5.379E-04/0.49			
	Learned_Regular	3.829E-01	1.927E-04/0.623		
	Unlearned_Irregular	9.337E-01	3.150E-03/0.487	4.073E-01	
	Unlearned_Regular	2.225E-01	4.554E-02/0.311	7.120E-02/0.426	3.247E-01
4	Learned_Irregular	1.820E-03/0.41			
	Learned_Regular	6.823E-01	2.301E-03/0.46		
	Unlearned_Irregular	5.540E-01	1.314E-03/0.512	8.770E-01	
	Unlearned_Regular	8.700E-01	9.761E-03/0.399	6.199E-01	5.130E-01
5	Learned_Irregular	2.250E-02/0.3			
	Learned_Regular	8.261E-01	3.045E-02/0.33		
	Unlearned_Irregular	5.949E-01	1.485E-02/0.383	7.890E-01	
	Unlearned_Regular	5.616E-01	1.339E-01	4.899E-01	3.357E-01

All possible pairs of conditions across subjects were compared on their mean of acquired visual sequences during each stage/run of encoding using t-test. The table showed all the p values of each t test between pairs. For significant difference, we included effect size after the p value. Red: $p<0.05$, Orange: $0.05<p<0.1$
