# Supplementary material for: Visual sequence encoding is modulated by music schematic structure and familiarity
Source: PLoS One. 2024 Aug 7;19(8):e0306271. doi: 10.1371/journal.pone.0306271 (PMC11305557; doi:10.1371/journal.pone.0306271)
Supplement: S7 Table — (PDF) [file pone.0306271.s007.pdf]

S7 Table

Accuracy ~ Familiarity x Regularity + (1|subject) excluding old-unlearned music trials
Analysis of Deviance Table (Type III Wald chi-square tests)

	df	*Chi-Square*	*P Value*
**Music Familiarity**	1	0.346	0.557
**Music Regularity**	2	7.134	0.028 *
**Familiarity x Regularity**	2	7.874	0.019 *

ResponseTime ~ Familiarity x Regularity + (1|subject) excluding old-unlearned music trials
Type III Analysis of Variance Table with Satterthwaite's method

	df	*Sum of Squares*	*Mean of Squares*	*F Value*	*P Value*
**Music Familiarity**	1	3.125	3.125	1.462	0.227
**Music Regularity**	2	35.902	17.951	8.402	0.0002***
**Familiarity x Regularity**	2	59.633	29.817	13.955	9.999E-07***

In our main analysis, we labeled music that was not unsuccessfully learned on Day1 as 'unlearned' music. We also ran supplementary analysis to exclude these samples and compare only 'new' music (music that only heard on Day2) versus 'learned' music (music that subjects successfully learned on Day1 and successfully recognized on Day2's first music recognition task). We used same linear mixed-effects models to test how music familiarity and music regularity predicted trial by trial visual sequences retrieval accuracy and response time. The tables showed the ANOVA results of the models. The results were similar to the main analysis shown in the paper. See Table 10 and 11 for pairwise comparison between conditions. **(p<0.001: ***, p<0.01: **, p <0.05: *, 0.05< p<0.1: ·)**
