# Supplementary material for: Visual sequence encoding is modulated by music schematic structure and familiarity
Source: PLoS One. 2024 Aug 7;19(8):e0306271. doi: 10.1371/journal.pone.0306271 (PMC11305557; doi:10.1371/journal.pone.0306271)
Supplement: S8 Table — (PDF) [file pone.0306271.s008.pdf]

S8 Table Post-hoc Tukey HSD test (accuracy) Excluding Old-Unlearned Trials

Comparison	*estimate*	*SE*	*z.ratio*	*p.value*
Familiar Control - Unfamiliar Control	-0.163	0.278	-0.588	0.992
Familiar Control - Familiar Irregular	0.529	0.291	1.82	0.452
Familiar Control - Unfamiliar Irregular	-0.733	0.311	-2.355	0.173
Familiar Control - Familiar Regular	-0.34	0.324	-1.05	0.901
Familiar Control - Unfamiliar Regular	-0.499	0.296	-1.687	0.54
Unfamiliar Control - Familiar Irregular	0.693	0.298	2.327	0.183
Unfamiliar Control - Unfamiliar Irregular	-0.57	0.317	-1.795	0.469
Unfamiliar Control - Familiar Regular	-0.176	0.33	-0.535	0.995
Unfamiliar Control - Unfamiliar Regular	-0.335	0.302	-1.111	0.877
Familiar Irregular - Unfamiliar Irregular	-1.262	0.33	-3.83	0.002
Familiar Irregular - Familiar Regular	-0.869	0.336	-2.585	0.101
Familiar Irregular - Unfamiliar Regular	-1.028	0.315	-3.267	0.014
Unfamiliar Irregular - Familiar Regular	0.393	0.358	1.098	0.882
Unfamiliar Irregular - Unfamiliar Regular	0.234	0.333	0.704	0.982
Familiar Regular - Unfamiliar Regular	-0.159	0.345	-0.461	0.997

The table showed all statistical results from the Tukey HSD test we conducted to follow up the significant interactive effect between music familiarity and music regularity on visual sequence retrieval accuracy. P value was adjusted and all significant pairs were highlighted. Red: $p < 0.05$
