# Supplementary material for: Visual sequence encoding is modulated by music schematic structure and familiarity
Source: PLoS One. 2024 Aug 7;19(8):e0306271. doi: 10.1371/journal.pone.0306271 (PMC11305557; doi:10.1371/journal.pone.0306271)
Supplement: S9 Table — (PDF) [file pone.0306271.s009.pdf]

S9 Table Post-hoc Tukey HSD test (RT) Excluding Old-Unlearned Trials

Comparison	*estimate*	*SE*	*df*	*t.ratio*	*p.value*
Familiar Control - Unfamiliar Control	0.086	0.128	1379.398	0.674	0.985
Familiar Control - Familiar Irregular	0.118	0.149	1384.557	0.788	0.970
Familiar Control - Unfamiliar Irregular	0.749	0.126	1378.795	5.927	0.000
Familiar Control - Familiar Regular	0.350	0.139	1381.691	2.525	0.118
Familiar Control - Unfamiliar Regular	-0.077	0.127	1378.807	-0.611	0.990
Unfamiliar Control - Familiar Irregular	0.032	0.149	1385.490	0.212	1.000
Unfamiliar Control - Unfamiliar Irregular	0.663	0.126	1378.895	5.265	0.000
Unfamiliar Control - Familiar Regular	0.264	0.138	1381.594	1.911	0.396
Unfamiliar Control - Unfamiliar Regular	-0.164	0.126	1379.001	-1.294	0.789
Familiar Irregular - Unfamiliar Irregular	0.631	0.148	1384.779	4.272	0.000
Familiar Irregular - Familiar Regular	0.232	0.157	1380.753	1.483	0.675
Familiar Irregular - Unfamiliar Regular	-0.195	0.148	1384.506	-1.317	0.776
Unfamiliar Irregular - Familiar Regular	-0.399	0.137	1380.990	-2.919	0.042
Unfamiliar Irregular - Unfamiliar Regular	-0.827	0.125	1378.544	-6.612	0.000
Familiar Regular - Unfamiliar Regular	-0.427	0.137	1381.285	-3.114	0.023

The table showed all statistical results from the Tukey HSD test we conducted to follow up the significant interactive effect between music familiarity and music regularity on response time for correctly retrieved visual sequences. P value was adjusted and all significant pairs were highlighted. Red: $p < 0.05$
