# Supplementary material for: Visual sequence encoding is modulated by music schematic structure and familiarity
Source: PLoS One. 2024 Aug 7;19(8):e0306271. doi: 10.1371/journal.pone.0306271 (PMC11305557; doi:10.1371/journal.pone.0306271)
Supplement: S10 Table — (PDF) [file pone.0306271.s010.pdf]

S10 Table

Accuracy ~ Familiarity x Regularity + (1|subject) **using all day 1 music as 'familiar' and never-heard before music as 'unfamiliar'**

Analysis of Deviance Table (Type III Wald chi-square tests)

	df	*Chi-Square*	*P Value*
**Music Familiarity**	1	10.204	0.209
**Music Regularity**	2	3.132	0.001 *
**Familiarity x Regularity**	2	5.198	0.074

ResponseTime ~ Familiarity x Regularity + (1|subject) **using all day 1 music as 'familiar' and never-heard before music as 'unfamiliar'**

	df	*Sum of Squares*	*Mean of Squares*	*F Value*	*P Value*
**Music Familiarity**	1	0.891	0.892	0.402	0.526
**Music Regularity**	2	17.401	8.7	3.924	0.019*
**Familiarity x Regularity**	2	40.904	20.452	9.223	1.043E-04***

In our main analysis, we labeled music that was not unsuccessfully learned on Day1 as 'unlearned' music. We also ran supplementary analysis to exclude these samples and compare only 'new' music (music that only heard on Day2) versus 'learned' music (music that subjects successfully learned on Day1 and successfully recognized on Day2's first music recognition task). We used same linear mixed-effects models to test how music familiarity and music regularity predicted trial by trial visual sequences retrieval accuracy and response time. The tables showed the ANOVA tables of the models using Car package in R. The results were similar to the main analysis shown in the paper. **(p<0.001: ***, p<0.01: **, p <0.05: *, 0.05< p<0.1: ·)**
