# Supplementary material for: Visual sequence encoding is modulated by music schematic structure and familiarity
Source: PLoS One. 2024 Aug 7;19(8):e0306271. doi: 10.1371/journal.pone.0306271 (PMC11305557; doi:10.1371/journal.pone.0306271)
Supplement: S1 Fig — (PDF) [file pone.0306271.s011.pdf]

S11 Figure Day 1 Training – Music Re-composition Task

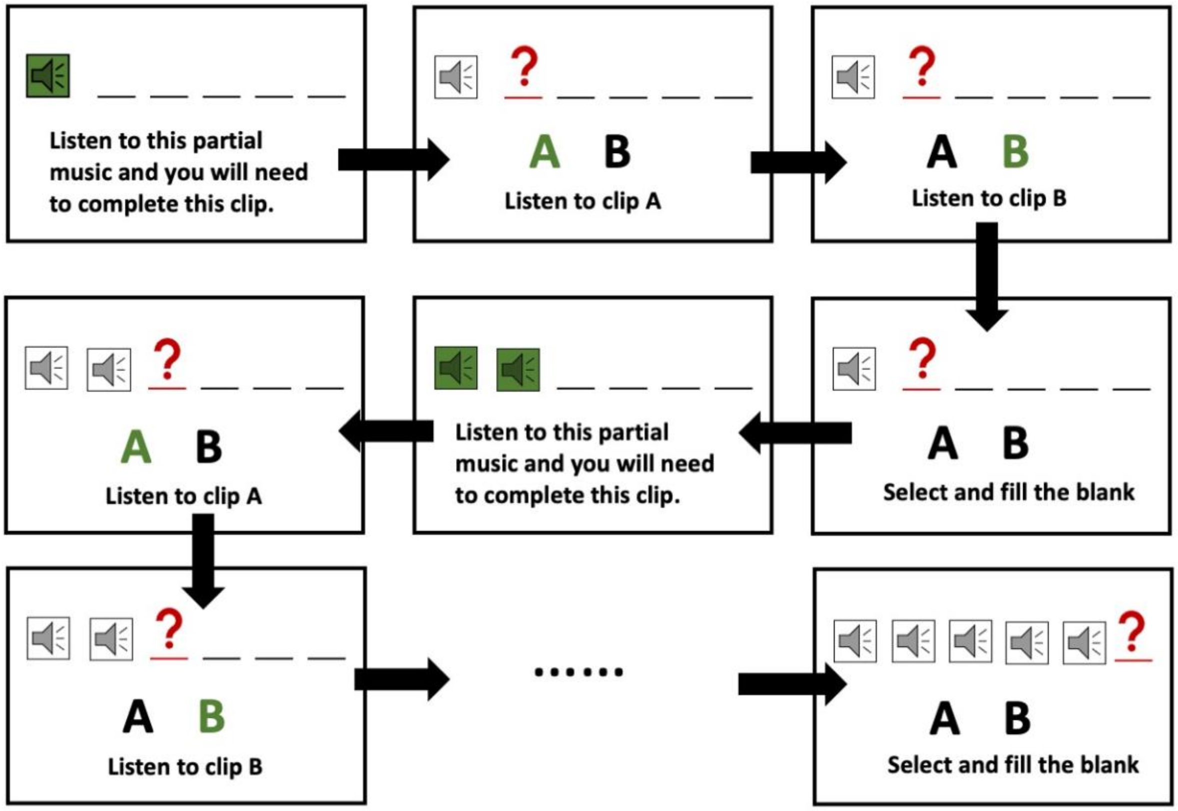

Day 1 music training paradigm: this music re-composition task was given after subjects listened to each music sitmuli with no time limits. The goal was to fully re-compose each music stimuli without errors. The task would not end untill subjects successfully re-composed every music clip correctly or they reached to the two hour time limits.
