# Supplementary material for: Visual sequence encoding is modulated by music schematic structure and familiarity
Source: PLoS One. 2024 Aug 7;19(8):e0306271. doi: 10.1371/journal.pone.0306271 (PMC11305557; doi:10.1371/journal.pone.0306271)
Supplement: S1 Text — (PDF) [file pone.0306271.s012.pdf]

***S12 Text***

***Visual Encoding and Individual Learning Profile Clustering***

In this study, we also examined how individual differences played a role in music's effects on learning. In a recent MRI study of ours (in a sample of all music-trained individuals), we noted differences in participants' feedback regarding whether and which music conditions helped them learn the best. Thus, in this behavioral study, collected a larger sample to ask if people with different traits learned visual sequences with concurrent music differently from each other.

With this particular interest, we ran a clustering analysis on the individuals' learning curve "profiles" – their pattern of learning curves across visual sequences paired with each condition of music (*learn-regular*, *learned-irregular*, *unlearned-regular*, *unlearned-irregular*, control). Thus, we used a K-means nearest neighbors algorithm and the 'elbow method' to select the most meaningful number of clusters. The features of each sample used were the participant's average visual sequence accuracies for all conditions across each visual sequence encoding run/phase. Five participants finished the task on Day 1 with 0 perfectly *learned-irregular* music - they therefore lacked features for the *learned-irregular* condition. Thus, the algorithm automatically dropped these five samples from this analysis, leaving us with 43 samples. We ran Pearson's chi-squared test with Monte Carlo simulation (due to the relatively small subgroup sample size) to further detect whether individual characteristics and music training background are associated with learning profiles/behaviors. For instance, does gender affect which learning profile an individual would present?

**Clustering Analysis of Visual Encoding Behaviors: Are there meaningful patterns in how different people learn with concurrent music?**

The overall learning profile (Fig.2.a) revealed no significant differences between conditions other than learned irregular music – nevertheless, it is of great interest that some people processed the music during learning more adeptly than others, and one possibility was that the average visual learning curves associated with different types of music encompassed multiple meaningful clusters of distinct individual learning profiles. To test this idea, we ran a KNN clustering analysis on peoples' condition*run visual learning curve patterns.

The KNN analysis uncovered 3 clusters ($n_1$= 18; $n_2$= 3; $n_3$= 22) of learning profiles during visual sequence encoding. We ran principal components analysis (PCA) and plotted out the clusters according to the first and second components representing the data, which explained 42.75% of the sample variability alone (Fig.2.b). Cluster 2 represented a more divergent behavior from the other two and was also quite small (n=3) (difficult to interpret or assess with inferential statistics). Therefore, we focused on Cluster #1 and #3 to investigate behavioral differences, running pair-wise t-tests to compare acquired sequences between conditions at each run. Because the purpose of this analysis was exploratory, to articulate where there was evidence of differences between conditions over learning in numerical terms, we report raw unadjusted p-values. In cluster #1 (Fig.2.c), differences from music conditions were restricted to the early stage of learning (run 1) with one main outcome: participants from this cluster learned the visual sequences paired with *learned-regular* the best and those paired with *unlearned-regular* the worst (*learned-regular* > *unlearned-regular*, $p = 0.025$; *unlearned-regular* < other conditions, $p = 0.06$-$0.08$, see details in Table 7). By contrast, cluster #3 participants (Fig.2.d) showed the most evident differences between *learned-regular* and *learned-irregular* – participants in this cluster

learned the visual sequences with *learned-irregular* music the slowest and with the lowest accuracy across run1-4 (*learned-irregular* < other condition, run 1-2 :p <0.001, run 3-4: p<0.05, see details in Table 8), while there were significant and statistical trends for *learned-regular* music benefitting visual sequence learning relative to the other conditions including the control in the middle stage of learning (*learned-regular* > other condition, run 2-3, p = 0.05-0.09) in addition to the differences relative to *learned irregular*.

In summary, participants in cluster 1 learned visual sequences differently only for the *regular* condition, with opposing performance relative to the other conditions (i.e., control and *irregular*) for *learned-regular* vs. *unlearned-regular* music. Cluster 3 participants showed an effect strongly aligned with our predictions: an opposite pattern for *regular* vs. *irregular* conditions that was dependent on the structure of each condition having been learned.

### 1.1.1 Different Learning Clusters and Individual Music Experiences Background

The clustering analysis revealed distinct patterns of visual learning that were affected by our paired music manipulation. The critical question was “why?”. We thus compared key participant variables based on the background literature such as age, gender and music preference for these two cluster distributions. We also revisited our theoretical interest in how music training might influence participant sensitivity to music structure and familiarity effects on learning.

We observed a trend that people with more music training background were less likely to reveal cluster 3 learning profiles (Fig 2.e) – subjects with experience with 0-1 instrument (combined for statistical power considerations) exhibited significantly different cluster membership than those who had systematically learned more than one instrument for at least a year (chi-squared = 3.90, bootstrap = 2000., simulated p = 0.031).

Mapping this result together with the learning characteristics of the clusters suggested that more training with music was associated with a greater sensitivity to whether concurrent *regular* music was learned or unlearned, and a lower likelihood that *learned-irregular* music would be disruptive to parallel learning. One speculation about our theoretical framework was that the subject who gained more music training would be more efficient at processing the music so that the retrieval of what tones, plus the paired shape, was coming next in learned music (even if *irregular*) was more automatic.

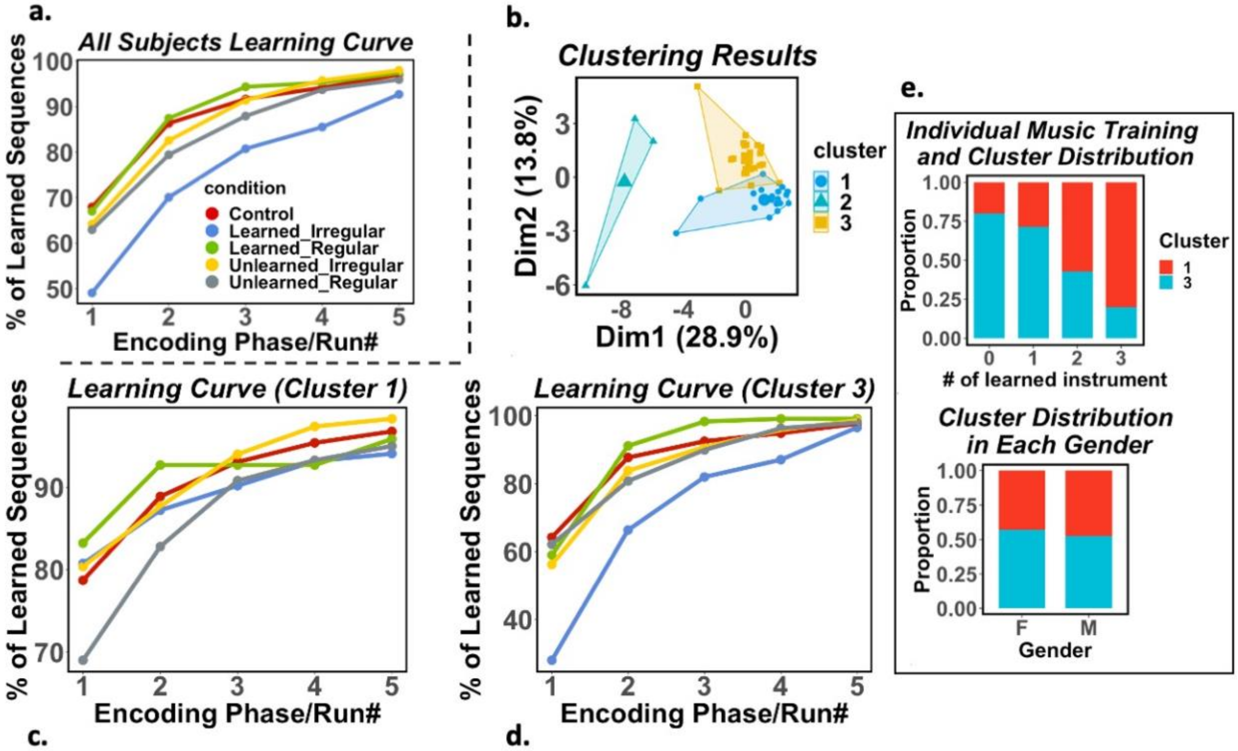


**Figure 2 Learning Profiles and Cluster Analysis**

**a)** This figure represented the cumulative learning curve during visual encoding for all subjects. The plot visualized the average proportion of visual sequences learned so far at each phase/run as a function of each music condition. The significantly slowest learning happened in the *learned-irregular* condition. **b)** PCA found the two most important components, and the plot represents cluster distribution based on these two values. **c) d)** Learning curve for averaged cumulative proportion of acquired sequences at each stage during visual sequence learning for cluster 1 (n=18) and 3 (n=22). **e)** cluster distribution on subjects grouped by music learning history. They showed that higher proportion of cluster 3 learning curves was associated with less music training. **Top**: grouped by years of music training history, comparing people who learned

instruments for less than two years versus those for more than two years. **Bottom**: grouped by how many instruments they had systematically learned for at least a year.
Note: learning curve for cluster 2 is not visualized due to the too-small sample size of the cluster for meaningful inference (n=3).

### 1.1.2 Applying Clusters from the Encoding Phase to Retrieval Results

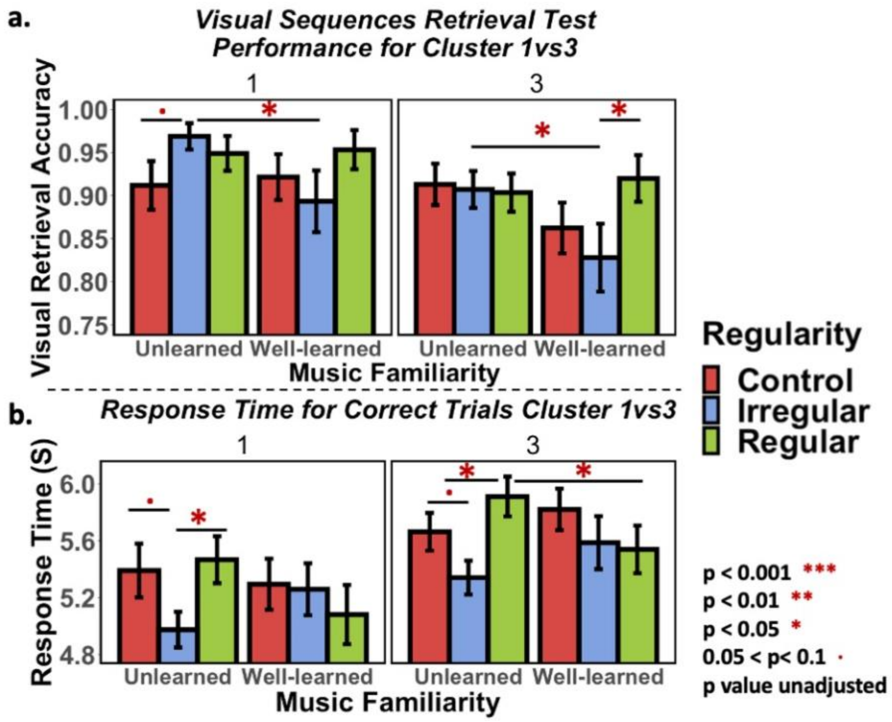


**Figure 3** Cluster 1 vs 3 Final Retrieval Performance

We then asked whether clusters of memory encoding were associated with different memory retrieval performances, especially between cluster 1 and cluster 3 with a trend that cluster 1 participant tended to have more music training background. Answering this question could be insightful for whether a specific learning pattern during encoding correlated to a better or worse final sequential memory performance. Fig.3 showed separate plots of visual retrieval accuracy and response time of correct trials for both clusters. First of all, interestingly, we found that

across conditions, compared with cluster 3, cluster 1's subjects (those with more music training experiences) achieved visual sequence retrieval with better accuracy ($t_{df=1431.9} = 2.994$, $p < 0.01$), and they generally retrieved correct trials with shorter response time across conditions ($t_{df=1204} = -4.4179$, $p<0.001$). Secondly, we ran pair-wise comparisons between conditions regarding their visual sequence retrieval accuracy and RT for correct trials. Due to the small sample size within clusters, we utilized an unadjusted pair-wise t-test to reveal the trending patterns in cluster differences that could be explored in future work. See Table 9 for statistical results. When comparing between clusters and referencing back to the whole-sample result, we found similar patterns in both clusters – the *learned-irregular* condition showed the worst retrieval accuracy. In contrast, the *learned-regular* condition showed a tendency for improved accuracy (it is challenging to interpret significance levels due to the small sample size). Interestingly, cluster 1 itself showed improved retrieval accuracy for *unlearned-irregular* music. RT for correct trials plot also showed similar patterns to the overall dataset – the *unlearned-irregular* music condition had shorter response times than the control, implying a stronger memory on correct trials than the control.
